# AMPK phosphosite profiling by label-free mass spectrometry reveals a multitude of mTORC1-regulated substrates

**DOI:** 10.1038/s44324-025-00052-7

**Published:** 2025-03-04

**Authors:** William J. Smiles, Ashley J. Ovens, Dingyi Yu, Naomi X. Y. Ling, Andrea C. Poblete Goycoolea, Kaitlin R. Morrison, Emmanuel O. Murphy, Astrid Glaser, Sophie F. Monks O’Byrne, Scott Taylor, Alistair M. Chalk, Carl R. Walkley, Luke M. McAloon, John W. Scott, Bruce E. Kemp, Ashfaqul Hoque, Christopher G. Langendorf, Janni Petersen, Sandra Galic, Jonathan S. Oakhill

**Affiliations:** 1 Metabolic Signalling Laboratory, St. Vincent’s Institute of Medical Research, Fitzroy, VIC 3065 Australia; 2 Research Program for Receptor Biochemistry and Tumour Metabolism, Department of Paediatrics, University Hospital of the Paracelsus Medical University, Salzburg, Austria; 3 Protein Engineering in Immunity and Metabolism, St. Vincent’s Institute of Medical Research, Fitzroy, VIC 3065 Australia; 4 Protein Chemistry and Metabolism, St. Vincent’s Institute of Medical Research, Fitzroy, VIC 3065 Australia; 5 Flinders Health and Medical Research Institute, Flinders Centre for Innovation in Cancer, Flinders University, Adelaide, SA 5042 Australia; 6 Genome Stability Unit, St. Vincent’s Institute of Medical Research, Fitzroy, VIC 3065 Australia; 7 Cancer and RNA Biology, St. Vincent’s Institute of Medical Research, Fitzroy, VIC 3065 Australia; 8 Department of Medicine, University of Melbourne, Parkville, VIC 3010 Australia; 9 Drug Discovery Biology, Monash Institute of Pharmaceutical Sciences, Parkville, VIC 3052 Australia; 10 Mary McKillop Institute for Health Research, Australian Catholic University, Melbourne, VIC 3000 Australia; 11 The Florey Institute of Neuroscience and Mental Health, Royal Parade, Parkville, VIC 3052 Australia

**Keywords:** Biochemistry, Biological techniques, Cancer

## Abstract

The nutrient-sensitive protein kinases AMPK and mTORC1 form a fundamental negative feedback loop that governs cell growth and proliferation. mTORC1 phosphorylates α2-S345 in the AMPK αβγ heterotrimer to suppress its activity and promote cell proliferation under nutrient stress conditions. Whether AMPK contains other functional mTORC1 substrates is unknown. Using mass spectrometry, we generated precise stoichiometry profiles of phosphorylation sites across all twelve AMPK complexes expressed in proliferating human cells and identified seven sites displaying sensitivity to pharmacological mTORC1 inhibition. These included the abundantly phosphorylated residues β1-S182 and β2-S184, which were confirmed as mTORC1 substrates on purified AMPK, and four residues in the unique γ2 N-terminal extension. β-S182/184 phosphorylation was elevated in α1-containing complexes relative to α2, an effect attributed to the α-subunit serine/threonine-rich loop. Mutation of β1-S182 to non-phosphorylatable Ala had no effect on basal and ligand-stimulated AMPK activity; however, β2-S184A mutation increased nuclear AMPK activity, enhanced cell proliferation under nutrient stress and altered expression of genes implicated in glucose metabolism and Akt signalling. Our results indicate that mTORC1 directly or indirectly phosphorylates multiple AMPK residues that may contribute to metabolic rewiring in cancerous cells.

## Introduction

Central to the regulation of cellular metabolism are signalling networks that couple the direct sensing of metabolites to metabolic flux and cell growth and proliferation through covalent modification of proteins, namely phosphorylation^1,2^. Fundamental to metabolic homoeostasis is the nutrient-sensitive Ser/Thr protein kinases AMP-activated protein kinase (AMPK) and mechanistic target of rapamycin complex 1 (mTORC1), which broadly speaking, antagonistically drive catabolic (e.g., autophagy, lipolysis) and anabolic (e.g., protein and ribosome synthesis) processes, respectively. AMPK is considered the energy guardian of the cell due to its role in maintaining cellular energy balance^3^.

The AMPK heterotrimeric complex comprises an α catalytic subunit and β and γ regulatory subunits^4^. Multiple isoforms of each subunit exist (α1/2, β1/2, γ1/2/3), allowing the formation of up to 12 unique AMPK complexes, each with distinct tissue expression patterns and biochemical properties^5^. The α-subunit possesses an archetypal kinase domain (α-KD) structure with an activation loop phosphorylation site (phosphosite) at T174 in α1 and T172 in α2 (collectively referred to as pT172) that is predominantly targeted by liver kinase B1 (LKB1) and the Ca^2+^/calmodulin-dependent protein kinase kinase 2 (CaMKK2)^6–9^. Basal pT172 stoichiometry in cultured mammalian HEK293T and COS7 cells has been independently estimated at 1–4%, rising to 8–10% following CaMKK2 activation or glucose starvation^10,11^, whereas another study estimated basal pT172 stoichiometry at 27% in HEK293 cells, rising to 50% in response to the potent mitochondrial respiratory chain inhibitor berberine^12^. COOH-terminal to the α-KD are regulatory elements including an autoinhibitory domain (AID), α-regulatory subunit-interacting motifs (α-RIM) that interact with the γ-subunit, a β-subunit interacting domain (β-SID) important for complex stability and a disordered Ser/Thr-rich loop heavily modified by phosphorylation (ST loop, α1 residues 471–530)^13^. ST loop phosphosites include α1-S487, a substrate for kinases in the AGC family such as Akt^13^, and the equivalent α2 autophosphorylation site S491^14,15^. The effect of ST loop phosphorylation is generally considered to limit AMPK activity by reducing net α-pT172^13^.

The β-subunit contains a mid-molecule carbohydrate-binding module (CBM) and a COOH-terminal, scaffolding α-γ-subunit binding sequence (αγ-SBS) that stabilises the heterotrimer^16^. The two regions are connected by a highly flexible loop of 22-24 residues called the β-linker. In AMPK crystal structures, a hydrophobic cleft termed the ADaM (Allosteric Drug and Metabolite) site forms between the CBM and the N-lobe of the α-KD, allowing for allosteric activation by long-chain fatty acyl-CoA esters (e.g., palmitoyl-CoA) and small synthetic compounds in a manner largely regulated by phosphorylation of the CBM residue, β1-S108^16–20^. β1-pS108 stoichiometries in cultured cells closely match those of α-pT172, in accordance with this residue being autophosphorylated, as well as a substrate for the autophagy master regulatory kinase, ULK1^10^.

The γ-subunit is formed by four cystathionine β-synthase repeats and contains exchangeable adenine nucleotide binding sites that allow AMPK to sense elevations in AMP/ATP and ADP/ATP ratios during periods of energy or nutrient stress (e.g., exercise, fasting, hypoxia)^12,21,22^. AMP binding is sensed by the α-RIM modules, which sequester the AID from the α-KD to allosterically activate AMPK. γ2 and γ3 isoforms contain non-conserved 259 (γ2) and 182 (γ3) amino acid NH_2_-terminal extensions (NTEs) of largely unknown function, although several phosphosites have been detected on the γ2-NTE (PhosphoSitePlus^®^^23^). Thus, in line with its central role in metabolic regulation, AMPK receives multidirectional signalling inputs via a range of regulatory phosphorylation sites present across each of its subunits^13^.

The kinase mTOR is the catalytic component of mTORC1 and mTORC2, two structurally and functionally distinct multi-protein complexes defined by unique regulatory partners that dictate substrate selectivity (mTORC1, Raptor; mTORC2, Rictor/SIN1). mTORC1 is activated by growth factors and nutrients such as glucose and amino acids, which canonically harness the lysosome as the cellular locale integrating these physiological cues to stimulate mTORC1^24–27^. Following activation, mTORC1 preferably phosphorylates Ser residues immediately NH_2_-terminal to a Pro residue (Ser/Thr-Pro sites). AMPK inhibits mTORC1 directly by phosphorylation of Raptor and indirectly by phosphorylation of TSC2 and the nutrient-sensing GATOR2 complex^28–31^. mTORC1 reciprocally inhibits AMPK activity in yeast and mammalian cells by directly phosphorylating the Ser-Pro sites α1-S347 and α2-S345, preventing association with LKB1 at the lysosome^32,33^. Other mTORC1 Ser-Pro substrates on AMPK have been identified by in vitro kinase assay, including α2-S377, β1-S182 and β2-S184, although the functional significance of their phosphorylation remains unknown^34^.

Examinations of cellular signalling have traditionally relied on semi-quantitative immunoblotting techniques (e.g. Western blotting) that carry significant limitations associated with signal detection linearity, the availability of high-quality phosphosite-specific antibodies, and lengthy nature of analysing multiple phosphosites. Although calculating phosphorylation stoichiometry through immunoblotting is possible with rigorous experiments using standards of known stoichiometry for each site, this is a laborious task that is not suitable for all phosphosites^10,35^. Mass spectrometry (MS)-based approaches have been used to accurately calculate stoichiometry of post-translational modifications using stable isotope standards or stable isotope labelling^36,37^, however these also require significant investment of time and resources that hinder high-throughput capability. Label-free MS techniques bypass these inconveniences and have successfully been used to calculate phosphorylation stoichiometries^38,39^. Here, we employ a highly targeted label-free MS-based approach to determine the basal phosphorylation stoichiometries of 19 phosphosites (10 novel, and 13 with unknown function) across all 12 AMPK complexes expressed in mammalian cells under full growth conditions. Notably, β1-S182 and the analogous β2 residue S184 are the most heavily phosphorylated sites on AMPK, and along with seven other phosphosites including four in the γ2-NTE, are sensitive to pharmacological mTOR inhibition in mammalian cells. We report that β1-S182 and β2-S184 are direct mTORC1 substrates and that loss of β2-pS184 increases nuclear AMPK activity, altering the expression of genes implicated in central carbon metabolism, and enhancing cell proliferation under nutrient stress. The β2-subunit isoform is frequently amplified in a range of cancers, and as such, these results offer key mechanistic insight into how cancerous cells could leverage AMPK activity to provide a growth and survival advantage when faced with a nutrient-poor tumour microenvironment^40^.

## Results

### Determining phosphorylation profiles across all AMPK heterotrimers

**Fig. 1:**
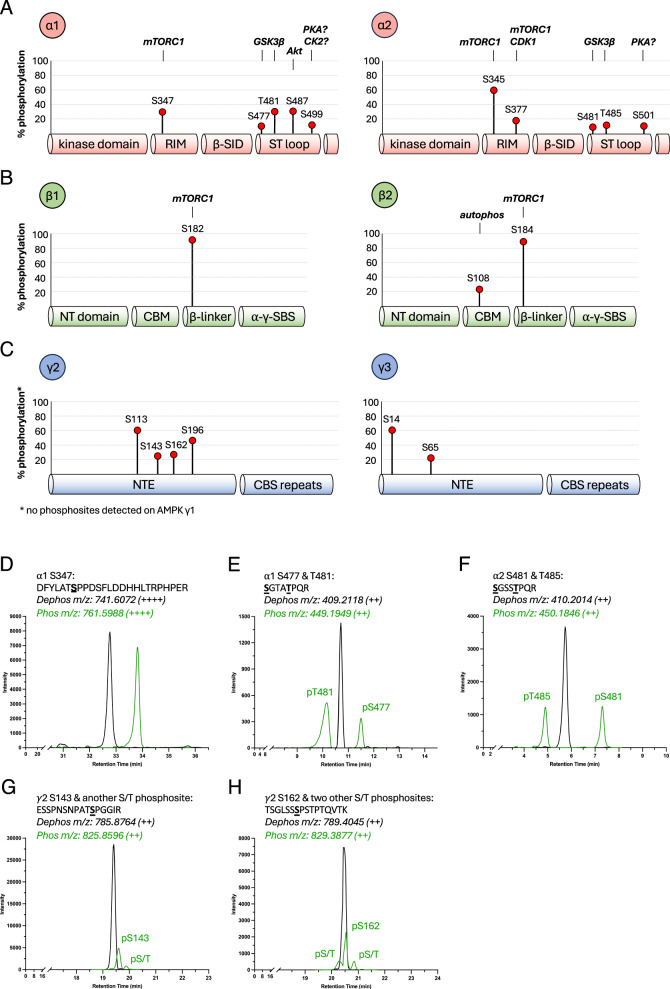
MS-detected AMPK phosphosites by subunit domain. All 12 AMPK complexes were FLAG-immunoprecipitated from HEK293T/17 cells in complete growth media and subjected to tryptic digest. Peptides and phosphopeptides from (**A**) α1 and α2, **B** β1 and β2, and (**C**) γ2 and γ3 were detected by LC-MS and area under the curve was used to calculate stoichiometries. Lengths of each marker line represent the average % stoichiometry from all complexes containing each isoform. Putative direct upstream kinases (not exhaustive) are displayed above each site where investigated. For β1-S182 and β2-S184 see Fig. 4. Note, some well-characterised AMPK phosphosites (α-pT172, β1-pS108) were not detected by this method for various reasons, as considered in main text. RIM: regulatory subunit-interacting motif; β-SID: β-subunit interacting domain; ST loop: Ser/Thr-rich loop; NT: NH_2_-terminal; CBM: carbohydrate binding module; α-γ-SBS: α-γ-subunit binding sequence; NTE: NH_2_-terminal extension; CBS: cystathionine β-synthase. Example extracted ion counts (EIC) for cognate phosphorylated and dephosphorylated peptides for (**D**) α1-S347, **E** α1-S477 and α1-T481, **F** α2-S481 and α2-T485, **G** γ2-S143 with another unidentified phosphosite, and (**H**) γ2-S162 with two unidentified phosphosites are shown.

To profile isoform-specific AMPK phosphosites, we expressed the twelve AMPK heterotrimer combinations as FLAG-fusions in the human cell line HEK293T/17. AMPK was FLAG-immunoprecipitated from cells grown in a complete medium, digested with trypsin and peptides analysed by liquid chromatography-mass spectrometry (LC-MS). A targeted approach was employed to achieve optimal sequence coverage of Ser/Thr residues on the seven AMPK isoforms (Table 1), from which we were able to quantify the stoichiometries of phosphosites in six isoforms using phosphorylated and dephosphorylated peptide peak areas (Eq. 1; Table 2; Fig. 1). The majority of phosphopeptides we detected contained a single phosphopeptide species (Fig. 1D; Table 2); however, there were instances of two (Fig. 1E–G) and three phosphopeptide species (Fig. 1H). Importantly, this did not hinder quantification since each of the phosphopeptide species possessed unique retention times. However, for peptides with two or three unique phosphospecies, very small quantities of di-phosphopeptide were sometimes detected. Since these were <10% of the signal of the mono-phosphopeptides this prevented quantification and were therefore excluded from the analysis. The identity of all sites was validated by MS/MS, and where multiple phosphopeptide species were present, these were further validated by site-directed mutagenesis (data not shown). This approach yielded precise quantification of 19 phosphosites, of which eleven (α1: S347, S477, T481, S487, S499; α2: S345, S377, T485; β1: S182; β2: S108, S184) have been identified by targeted methods, six (α2: S481; γ2: S113, S143, S162, S196; γ3: S65) have been identified in high-throughput studies, and two (α2: S501; γ3: S14) appear novel (Fig. 1A–C; Table 3)^13,23^. Phosphorylation stoichiometries of these sites ranged from ~4% (α2-pS481 in α2β1γ1) to ~96% (β-pS182/184 in α2β1γ1 and α1β2γ1).

**Table 1:** Tryptic peptide coverage of AMPK isoforms analysed in Fig. 1

Sequence coverage		Phosphosite(s)
Start	End	
**α1 (1-550): 51% coverage**		
V13	K31	–
S56	K62	–
D141	R173	–
H249	K276	–
N317	R334	–
D341	K387	S347
S404	R417	–
Q421	R435	–
K439	R492	S477, T481, S487
S497	K546	S499
**α2 (1-552): 53% coverage**		
V11	K41	–
S54	K60	–
D139	R171	–
I226	R256	–
D261	K269	–
I333	R381	S345, S377
A400	R421	–
Q425	R439	–
R442	R463	–
S471	R488	S481, T485
S500	R552	S501
**β1 (1-270): 59% coverage**		
A73	K102	–
S108	K217	S182
β2 (1-272): 56% coverage		
I35	K167	S108
D180	R197	S184
**γ2 (1-569): 51% coverage**		
V35	K57	–
K62	K96	–
T104	R148	S113, S143
K155	K171	S162
I191	R203	S196
A228	R263	–
L280	K311	–
Q423	K510	–
**γ3 (1-489): 33% coverage**		
T12	R48	S14
Q63	K95	S65
L170	R186	–
A215	K233	–
T347	R379	–
R399	R422	–

**Table 2:** Cognate peptide and phosphopeptides detected by LC-MS on AMPK expressed in HEK293T/17 cells under complete growth conditions

Phosphosite	Cognate peptide and phosphopeptide	Precursor *m/z* (charge)	Charges used	Flyability ratio
**α1 (1-550)**				
S347	Dephos: DFYLATSPPDSFLDDHHLTRPHPER	741.6072 (++++)	++++	0.58
	Phos: DFYLATS[+80]PPDSFLDDHHLTRPHPER	761.5988 (++++)		
S477	Dephos: SGTATPQR	409.2118 (++)	++	1.30
	Phos: S[+80]GTATPQR	449.1949 (++)		
T481	Dephos: SGTATPQR	409.2118 (++)	++	1.11
	Phos: SGTAT[+80]PQR	449.1949 (++)		
S487	Dephos: SGSVSNYR	435.2092 (++)	+, ++	1.10
	Phos: SGS[+80]VSNYR	475.1924 (++)		
S499	Dephos: SDSDAEAQGK	504.2174 (++)	+, ++	0.98
	Phos: SDS[+80]DAEAQGK	544.2006 (++)		
**α2 (1-552)**				
S345	Dephos: IMNQASEFYLASSPPSGSFMDDSAMHIPPGLKPHPER	809.1845 (5+)	+++++	0.83
	Phos: IMNQASEFYLASS[+80]PPSGSFMDDSAMHIPPGLKPHPER	825.1777 (5+)		
S377	Dephos: MPPLIADSPK	534.7915 (++)	+, ++	0.89
	Phos: MPPLIADS[+80]PK	574.7747 (++)		
S481	Dephos: SGSSTPQR	410.2014 (++)	+, ++	1.35
	Phos: S[+80]GSSTPQR	450.1846 (++)		
T485	Dephos: SGSSTPQR	410.2014 (++)	+, ++	1.18
	Phos: SGSST[+80]PQR	450.1846 (++)		
S501	Dephos: SSFDSTTAESHSLSGSLTGSLTGSTLSSVSPR	1048.5040 (+++)	+++, ++++	1.56
	Phos: SS[+80]FDSTTAESHSLSGSLTGSLTGSTLSSVSPR	1075.1595 (+++)		
**β1 (1-270)**				
S182	Dephos: C[−1]SDVSELSSSPPGPYHQEPYVC[−1]KPEER	755.3366 (++++)	+++, ++++	1.31
	Phos: C[−1]SDVSELSSS[+80]PPGPYHQEPYVC[−1]KPEER	775.3281 (++++)		
**β2 (1-272)**				
S108	Dephos: SHNDFVAILDLPEGEHQYK	553.7722 (++++)	+++, ++++	2.97
	Phos: S[+80]HNDFVAILDLPEGEHQYK	573.7638 (++++)		
S184	Dephos: DLSSSPPGPYGQEMYAFR	667.9719 (+++)	+++	1.02
	Phos: DLSSS[+80]PPGPYGQEMYAFR	694.6273 (+++)		
**γ2 (1-569)**				
S113	Dephos: TVFPFSYQESPPR	777.8830 (++)	++, +++	5.11
	Phos: TVFPFSYQES[+80]PPR	817.8662 (++)		
S143	Dephos: ESSPNSNPATSPGGIR	785.8764 (++)	++, +++	1.87
	Phos: ESSPNSNPATS[+80]PGGIR	825.8596 (++)		
S162	Dephos: TSGLSSSPSTPTQVTK	789.4045 (++)	++, +++	2.79
	Phos: TSGLSSS[+80]PSTPTQVTK	829.3877 (++)		
S196	Dephos: IYASSSPPDTGQR	689.8335 (++)	++, +++, ++++	0.71
	Phos: IYASSS[+80]PPDTGQR	729.8167 (++)		
**γ3 (1-489)**				
S14	Dephos: TPSWSSLGGSEHQEMSFLEQENSSSWPSPAVTSSSER	1007.1987 (++++)	+++, ++++	0.99
	Phos: TPS[+80]WSSLGGSEHQEMSFLEQENSSSWPSPAVTSSSER	1027.1903 (++++)		
S65	Dephos: SVEEGEPPGQGEGPR	762.8499 (++)	++	12.22
	Phos: S[+80]VEEGEPPGQGEGPR	802.8330 (++)		

Mass/charge (*m/z*) for the dominant charge state (shown in parentheses) are shown here as well as the charge states used for the calculation of stoichiometries. The flyability ratio (*k)* for each phosphopeptide is also displayed.

**Table 3:**
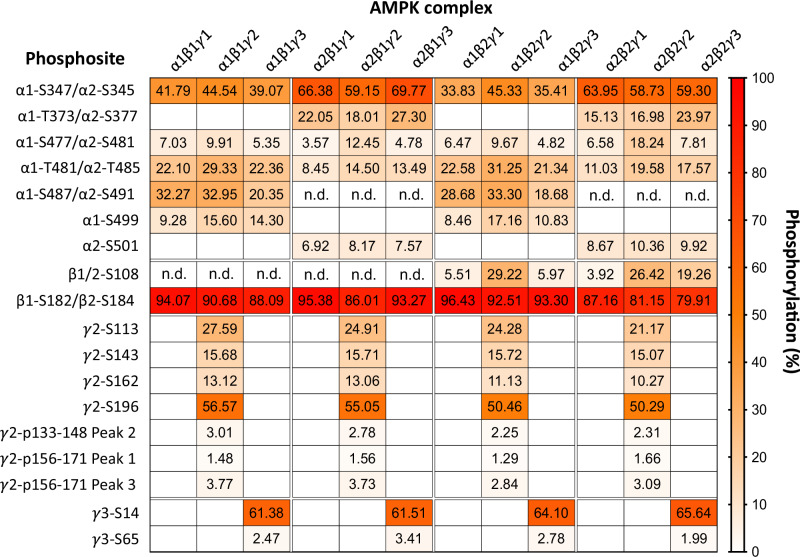
LC-MS determined stoichiometries of phosphosites identified on AMPK expressed in HEK293T/17 cells under complete growth conditions

Phosphosites are paired according to the conservation between isoforms. Values represent average stoichiometries from three replicates. SEM values ranged from 0.09 (α2-pT485 on α2β1γ3) to 5.40 (γ3-pS65 on α2β1γ3. N.B. PhosphoSitePlus displays α1 phosphosite numbering based on the longer recognised α1 variant (+9 residues at NH_2_-terminus).

**Fig. 2:**
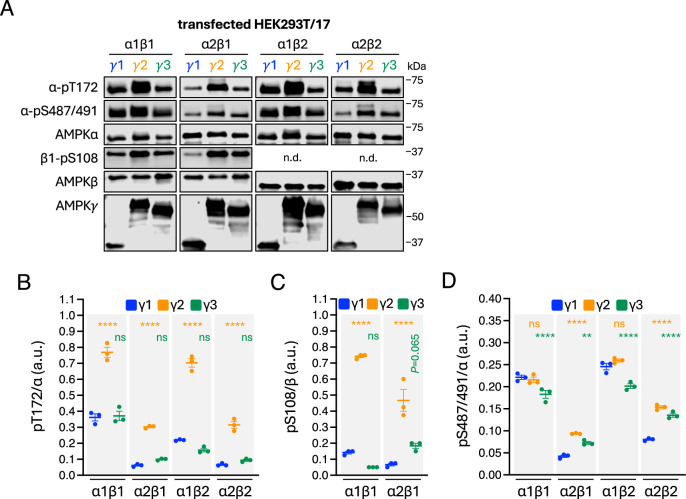
Basal comparison of regulatory phosphosites across 12 AMPK complexes. **A** AMPK complexes were FLAG-immunoprecipitated from HEK293T/17 cells incubated in complete growth media and immunoblotted as indicated. Representative immunoblots from 3 independent experiments are shown. Quantitation of (**B**) α-pT172, **C** β1-pS108, and (**D**) α-pS487. Data presented as mean phosphorylation (a.u. arbitrary units) ± SEM, *n* = 3. Statistical analyses were performed by one-way ANOVA with Dunnett’s multiple-comparisons test. **P < 0.01, ****P < 0.0001 vs. respective γ1 complex. n.s. not significant; n.d. not determined.

This MS approach did not detect well-known AMPK phosphosites α-pT172, α2-pS491 and β1-pS108, likely due to impeded tryptic cleavage or interfering mass species eluting at similar retention times^41,42^. Instead, these phosphosites were detected on FLAG-immunoprecipitated AMPK by Western blotting using validated, phosphosite-specific antibodies (Fig. 2A). Activation loop α-T172, whose phospho-antibody recognition epitope is fully conserved between α1 and α2, was more heavily phosphorylated in α1 than α2 (Fig. 2B), which is consistent with AMPK purified from rat liver^6^. α2-pT172 has been shown to be more sensitive to phosphatases than α1-pT172, suggesting greater turnover in mammalian cells^43^. α-T172 was also more heavily phosphorylated in γ2-complexes than in corresponding γ1- and γ3-complexes (Fig. 2B), possibly due to a role for the γ2-NTE in protecting α-pT172 from dephosphorylation by phosphatases^44,45^. Consistent with higher α-pT172 and autophosphorylation activity in γ2-AMPK, β1-pS108 and β2-pS108 (detected by immunoblot and LC-MS, respectively) were significantly elevated in γ2-complexes compared to γ1- and γ3-complexes (Fig. 2A, C; Table 3).

### ST loop phosphorylation profiles

We detected seven phosphosites in the highly regulated α-ST loop. α1-pS487, but not the analogous α2-pS491, was quantified at stoichiometries between 20-35% depending on the γ isoform (Table 3), with the lowest stoichiometries detected in γ3-AMPK. The inability to detect α2-pS491 by LC-MS may stem from the α2-specific residue P498 blocking tryptic cleavage, as we could detect the analogous mouse α2 peptide harbouring a natural P498A substitution, which was approximately 20% phosphorylated on γ1-AMPK (data not shown). Immunoblots for α1-pS487 and α2-pS491 using a phospho-antibody that detects both sites supported the LC-MS data by demonstrating γ3-AMPK complexes have lower levels of α1-pS487, whereas γ2- and γ3-complexes display higher levels of α2-pS491 (Fig. 2D). While α1-S487 appeared to be more highly phosphorylated than α2-S491, we cannot rule out differences in antibody binding affinity between the 2 phosphosites as the epitope is not 100% conserved. We also detected ST loop phosphosites α1-pS477/α2-pS481 and α1-pT481/α2-pT485 (Fig. 1E, F) that were previously identified as GSK3β substrates^46^. Basal levels of α1-pS477/α2-pS481 were relatively consistent across the different AMPK combinations (4-22%), with stoichiometries again higher in γ2-AMPK, in particular α2γ2-containing complexes (Table 3). α1-pT481/α2-pT485 levels (10-32%) were generally higher in α1-AMPK and the highest when complexed with γ2. Since γ2-complexes possessed both the highest level of ST loop *and* α-T172 phosphorylation, this suggests the effect of the ST loop to attenuate α-pT172 may be context-dependent, or the γ2-NTE effect of preserving α-pT172 dominates.

We identified two uncharacterised ST loop phosphosites, α1-S499 and α2-S501, with α1-pS499 stoichiometries (8–17%) lowest in γ1-complexes and α2-pS501 stoichiometries (10–15%) consistent between AMPK heterotrimers. α1-S499 was previously shown to be phosphorylated in vitro by PKA^47^, despite this site being flanked by several acidic residues that point to a casein kinase 2 recognition motif^48^. The sequence surrounding α2-S501 (RPRSsFDST) does, however, appear to be a better fit for a PKA consensus motif (R/K-R/K-X-S/T, where X is any amino acid)^49^.

### Ser-Pro phosphorylation profiles

**Fig. 3:**
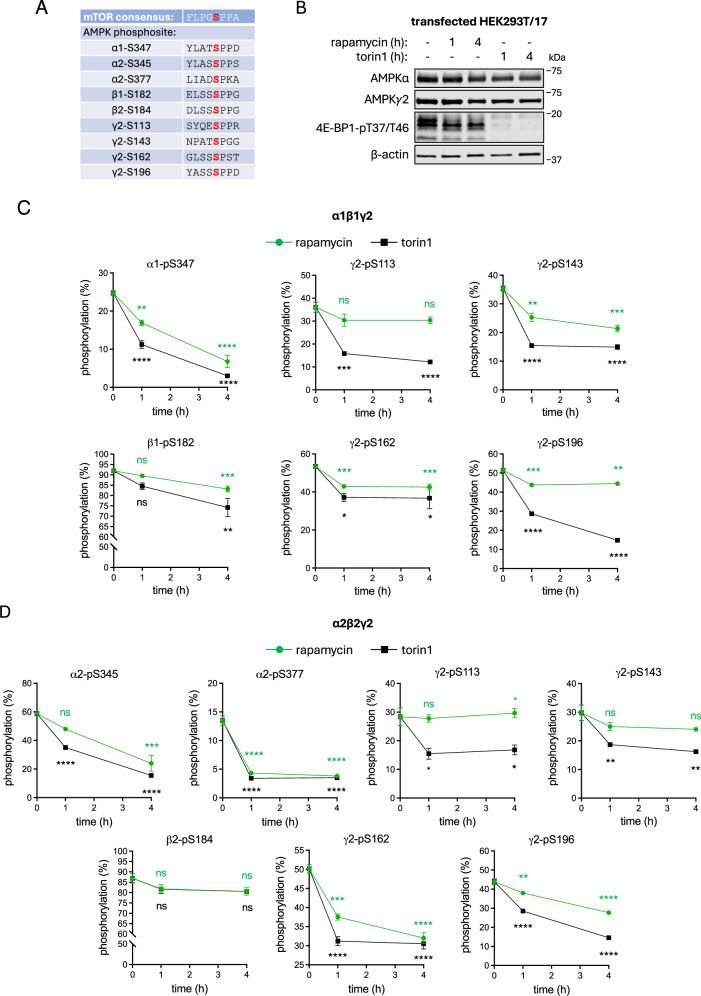
AMPK phosphosites sensitive to pharmacological inhibition of mTORC1. **A** Sequence alignment of selected Ser-Pro phosphosites on AMPK with the mTOR consensus motif^23^. **B** HEK293T/17 cells expressing α1β1γ2 or α2β2γ2 were treated with rapamycin (100 nM) or torin1 (1 µM) for up to 4 h and lysates immunoblotted as indicated. Representative immunoblots from 3 independent experiments are shown. **C** α1β1γ2 and (**D**) α2β2γ2 complexes from (**B**) were FLAG-immunoprecipitated and subjected to tryptic digest. Peptides and phosphopeptides were detected by LC-MS and area under the curve was used to calculate stoichiometries**;** rapamycin and/or torin1 sensitive phosphosites at 1 h and 4 h post-treatment are depicted here. Data presented as mean % stoichiometry ± SEM, *n* = 3. Statistical analyses were performed by one-way ANOVA with Dunnett’s multiple-comparisons test. *P < 0.05, **P < 0.01, ***P < 0.001, ****P < 0.0001 vs. basal. n.s. not significant.

Our targeted LC-MS analysis detected several Ser-Pro phosphosites in AMPK including the inhibitory mTORC1 substrates α1-pS347 and α2-pS345 (Fig. 3A). Stoichiometries for α2-pS345 (55-66%) were considerably higher than α1-pS347 (23-32%) and both showed little variation between β- and γ-subunit combinations (Table 3). α2-S377, an in vitro mTORC1 substrate and exercise-, insulin- and rapamycin-sensitive phosphosite detected in human skeletal muscle^34,50^, was comparably phosphorylated in γ1 and γ2 complexes (14-20% stoichiometries), with slightly higher stoichiometries in γ3-AMPK (22-25%; Table 3). We could not detect basal phosphorylation of the α1-equivalent residue T373 despite strong conservation with α2-S377 in the surrounding sequence and successful detection of the dephosphorylated peptide (data not shown). The largely uncharacterised Ser-Pro sites β1-S182 and β2-S184 in the β-linker connecting β-CBM and α-γ-subunit binding sequence were the most abundantly phosphorylated residues of all detected sites with near-maximal phosphorylation observed in basal state γ1-complexes (Fig. 1A–C; Table 3). β2-pS184 stoichiometries were generally lower in α2β2-complexes, although stoichiometries in all AMPK complexes were at 80% or above. For α2β2γ3 we have seen this site as low as 62% in an independent experiment. These data corroborate studies showing β1-S182 is stoichiometrically phosphorylated in largely α1-AMPK extracted from rat liver^51^, whereas β2-S184 is sub-stoichiometrically phosphorylated in rat skeletal muscle that predominantly contains α2β2-complexes^52^.

We detected four Ser-Pro sites in the γ2-NTE with basal stoichiometries between 24-66% (Table 3). γ2-pS113 was detected with stoichiometries over 57% in all γ2-complexes, and γ2-pS143 (~25%), γ2-pS162 (24-30%) and γ2-pS196 (42-68%) all contribute significantly to create a rich diversity of phosphorylation patterning in this region. We were unable to confidently assign or quantify three phosphosites in two Ser/Thr-rich phosphorylated peptides due to low levels of phosphorylation (133–148: ESSPNSNPATSPGGIR, Fig. 1G; 156–171: TSGLSSSPSTPTQVTK, Fig. 1H). We also identified two phosphosites on the γ3-NTE, S14 (>61% stoichiometry) and S65 (20-29%), with neither differentially phosphorylated between αβ combinations (Table 3). Phosphorylation of a γ3 peptide containing S14 and S16 has been reported although the precise phosphosite was not determined^53^. γ3-pS65 has been detected in human muscle but is not regulated by exercise^54^. We validated both sites with MS/MS and site-directed mutagenesis (data not shown).

### mTORC1 mediates the phosphorylation of multiple residues on AMPK

Capitalising on our all-in-one targeted LC-MS approach, we investigated which Ser-Pro phosphosites on AMPK might be regulated by mTORC1. Rapamycin, an incomplete but mTORC1-specific inhibitor, and torin1, a more efficient ATP-competitive inhibitor of mTOR, induced partial or complete dephosphorylation of the mTORC1 substrate 4E-BP1-pT37/46 in HEK293T/17 cells, respectively, indicating appropriate mTORC1 responsiveness to these compounds in this cell line (Fig. 3B)^55^. In the α1β1γ2 AMPK complex, our LC-MS method demonstrated that α1-pS347 was dephosphorylated from 25% basal stoichiometry to 17% and 7% following 1 h and 4 h of rapamycin treatment, respectively (Fig. 3C). α1-pS347 was dephosphorylated more robustly by torin1, reducing from 25% basal stoichiometry to 11% by 1 h and 3% by 4 h. β1-pS182, γ2-pS143, γ2-pS162 and γ2-pS196 were all significantly dephosphorylated compared to baseline after 4 h incubation with torin1, although even after this time approximately 75% of AMPK complexes remained phosphorylated at β1-S182. γ2-S113 phosphorylation levels were sensitive to torin1 but not rapamycin (Fig. 3C), a feature of several mTORC1 substrates (Fig. 3B)^55^. In the α2β2γ2 AMPK complex, α2-pS345 was partially dephosphorylated following 4 h incubation with rapamycin or torin1 (dropping from 59% stoichiometry to 24% or 16%, respectively), as previously described^33^, whereas α2-pS377, γ2-pS162 and γ2-pS196 were significantly dephosphorylated after 1 h incubation with both inhibitors (Fig. 3D). γ2-pS113 and γ2-pS143 were sensitive to torin1 treatment but not rapamycin (Fig. 3D). The β2-pS184 signal showed a downward trend compared to basal with both rapamycin and torin1 at 1 h and 4 h, although this change did not reach statistical significance (4 h torin1: *P* = 0.091).

### The α-ST loop moderates mTORC1-mediated phosphorylation of AMPK β-S182/184

**Fig. 4:**
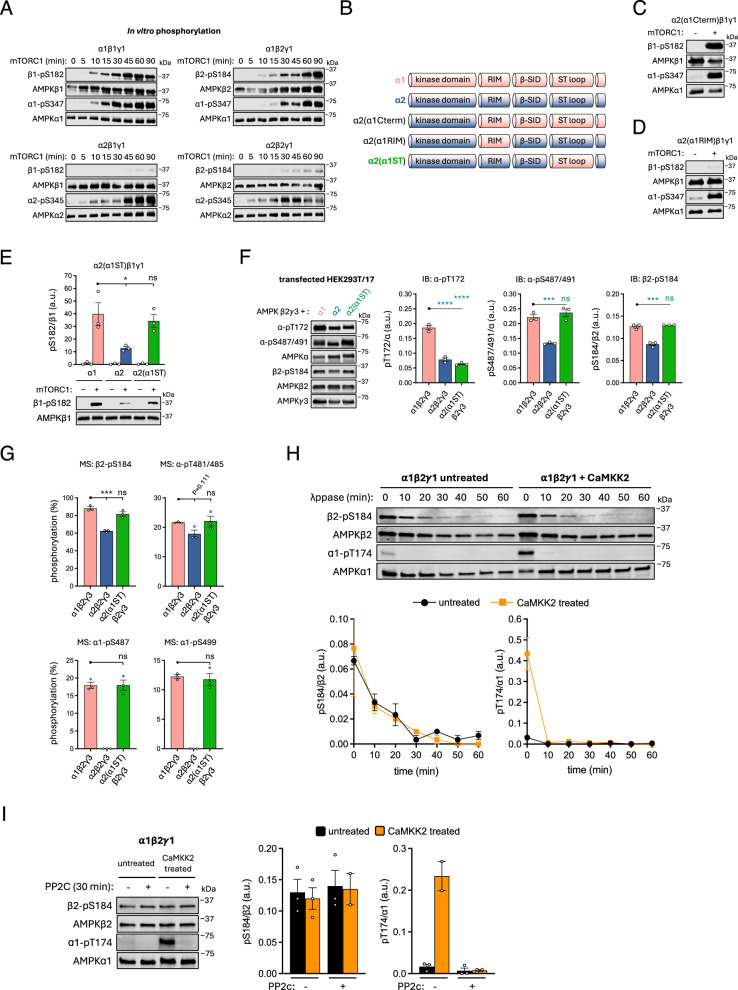
The AMPK α2-ST loop blocks mTORC1 phosphorylation of β-S182/184 and is associated with reduced cellular β2-pS184. **A** All four γ1-AMPK complexes were purified from *E. coli* as kinase-dead mutants (α1-D141A, α2-D139A), phosphorylated by mTORC1 in vitro for up to 90 min and immunoblotted as indicated. **B** Representation of α2/α1 chimeras. β1γ1, complexed to α1, α2 or (**C**) α2(α1Cterm), (**D**) α2(α1RIM) or (**E**) (α2(α1ST) chimeras were purified from *E. coli* as kinase-dead mutants (α1-D141A, α2-D139A), phosphorylated by mTORC1 in vitro for 30 min and β1-pS182 measured by immunoblot with α1-pS347 or α2-pS345 used as positive controls. For (**E**), data are presented as mean β1-S182 phosphorylation (a.u. arbitrary units) ± SEM, *n* = 3. Statistical analyses were performed by one-way ANOVA with Dunnett’s multiple-comparisons test. *P < 0.05. n.s. not significant. **F** Lysates were prepared from HEK293T/17 cells expressing β2γ3 complexed to α1, α2 or the α2(α1ST) chimera, AMPK complexes were FLAG-immunoprecipitated and immunoblotted for α-pS487/491, α-pT172, and β2-pS184. Data presented as mean phosphorylation (a.u. arbitrary units) ± SEM, *n* = 3. Statistical analyses were performed by one-way ANOVA with Dunnett’s multiple-comparisons test. ***P < 0.001, ****P < 0.0001. n.s. not significant. **G** AMPK complexes from (**F**) were subjected to tryptic digest. Peptides and phosphopeptides were detected by LC-MS and area under the curve used to calculate stoichiometries of β2-pS184 and ST loop phosphosites. Data presented as mean % stoichiometry ± SEM, *n* = 3. Statistical analyses were performed by one-way ANOVA with Dunnett’s multiple-comparisons test. ***P < 0.001, ****P < 0.0001. n.s. not significant. **H** In vitro time-course for lambda phosphatase (λppase) -mediated dephosphorylation of HEK293T-expressed and FLAG-purified α1β2γ1, with or without CaMKK2 pre-treatment (1:10 AMPK:phosphatase mass ratio). **I** In vitro, PP2C phosphatase-mediated dephosphorylation of HEK293T-expressed and FLAG-purified α1β2γ1 (untreated and CaMKK2 treated; 1:1 AMPK:phosphatase mass ratio). For (**H**, **I)**, AMPK was immunoblotted for α1-pT174 and β2-pS184, with quantitation presented as mean phosphorylation (a.u. arbitrary units) ± SEM, *n* = 2–3. Representative immunoblots from three independent experiments are shown.

We performed time course in vitro phosphorylation experiments using purified mTORC1 and bacterially-expressed, kinase-inactive AMPK γ1-complexes (α1-D139A or α2-D141A mutants to exclude AMPK autophosphorylation^10^). mTORC1 phosphorylated both β1-S182 and β2-S184, detected by immunoblot, with the rate of phosphorylation of both apparently much more rapid in α1- than α2-complexes (Fig. 4A). Conversely, mTORC1-mediated phosphorylation of α1-S347 and α2-S345 displayed comparable kinetics across the four γ1-complexes. To determine which region(s) of the α2 isoform is responsible for suppressing in vitro mTORC1-mediated phosphorylation of β1-S182, we generated a series of bacterially-expressed α2β1γ1 chimera complexes in which the α2 COOH-terminus (residues α2: 269-end), α2-RIM (α2: 348-396) or α2-ST loop (α2:475-532) sequences were individually exchanged for analogous α1 sequences (Fig. 4B). β2-S184 remained a poor mTORC1 substrate in the α2(α1RIM) chimera, whereas substantial phosphorylation under the same conditions was recovered in both α2(α1Cterm) and α2(α1ST) chimeras (Fig. 4C–E).

We repeated immunoblot and LC-MS analyses of HEK293T/17-expressed α1β2γ3 and α2β2γ3 and confirmed reduced basal β2-pS184 in the α2 complex relative to α1, in these preparations observing 62% and 88% stoichiometries, respectively (Fig. 4F, G). Expression of the α2(α1ST)β2γ3 chimera in HEK293T/17 cells resulted in complexes with basal phosphorylation of α1-ST loop residues S487 (measured by immunoblot and LC-MS), and T485, S487 and S499 (LC-MS) at similar levels to α1β2γ3, indicating appropriate cellular regulation and α1-ST loop modification in the α2(α1ST) chimera (Fig. 4F, G). Basal levels of α2-pT172 were similar between α2(α1ST)β2γ3 and α2β2γ3 complexes (immunoblot), indicating that the α2-ST loop was not entirely responsible for reduced basal pT172 seen in α2 versus α1. Conversely, basal β2-pS184 in α2(α1ST)β2γ3 was significantly elevated compared to α2β2γ3 (immunoblot and LC-MS), reaching levels comparable to α1β2γ3 (Fig. 4F, G). Combined, these data indicate that either the α2-ST loop impedes, or the α1-ST loop promotes, accessibility of mTORC1 to its AMPK substrate β-S182/184.

We reasoned that the apparent recalcitrance of β1-pS182 to dephosphorylation after 4 h of mTORC1 inhibition by torin1 (Fig. 4C) may be due to either upregulation of compensatory signalling pathways and/or restricted phosphatase accessibility to this site. In support of the latter, incubation of mammalian cell-expressed α1β2γ1 with lambda phosphatase resulted in a slower rate of dephosphorylation of β2-pS184 compared with α1-pT174, even when the complex was pre-treated with CaMKK2 to increase α1-pT174 by ~13-fold to match β2-pS184 stoichiometry (Fig. 4H). CaMKK2 pre-treatment had no effect on the rate of β2-pS184 dephosphorylation. A similar experiment performed with PP2C phosphatase resulted in complete loss of α1-pT174 following a 30 min incubation without affecting β2-pS184 (Fig. 4I). These data suggest the high cellular stoichiometries and low dephosphorylation rates of β-pS182/184 both *in cellulo* in response to mTOR inhibitors and during in vitro phosphatase assays, may at least in part be due to low phosphatase accessibility to these sites.

### β-S182/184 is likely a substrate in mTORC1-dependent and -independent signalling pathways

**Fig. 5:**
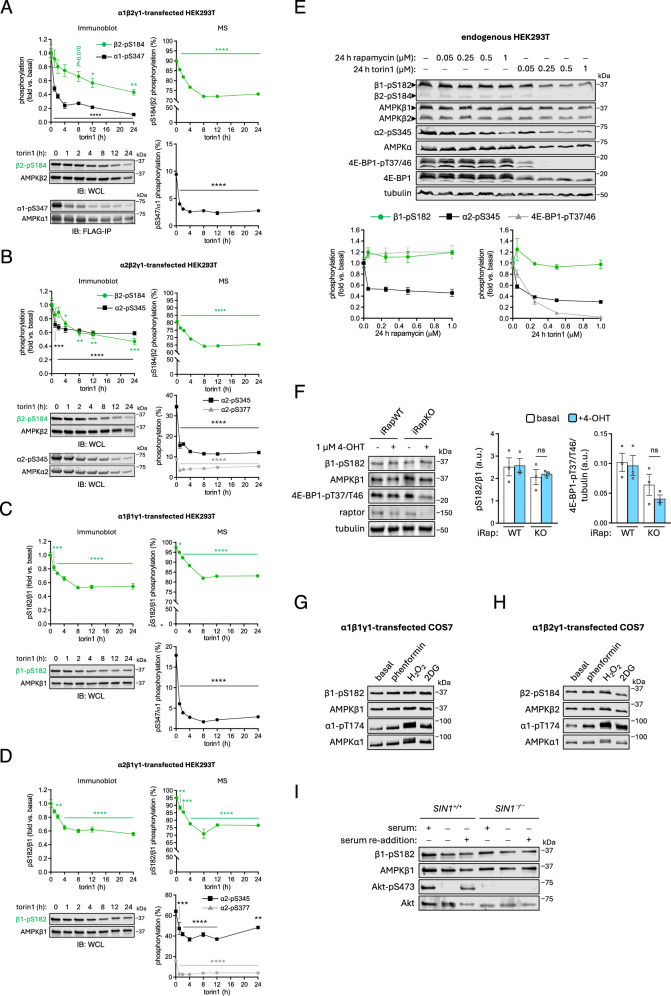
Longer exposure of HEK293T cells to torin1 leads to gradual loss of β-pS182/184 underpinned by slow in vitro dephosphorylation kinetics. HEK293T cells expressing FLAG-fusions of (**A**) α1β2γ1, (**B**) α2β2γ1, (**C**) α1β1γ1, or (**D**) α2β1γ1 were incubated with 250 nM torin1 for up to 24 h, and indicated phosphosites/stoichiometries detected from prepared lysates (WCL) or FLAG-immunoprecipitated AMPK by immunoblot or LC-MS. Data presented as mean phosphorylation (fold vs. baseline (0 h)) ± SEM, or mean % stoichiometry ± SEM, *n* = 3. Statistical analyses were performed by one-way ANOVA with Dunnett’s multiple-comparisons test. *P < 0.05, **P < 0.01, ***P < 0.001 and ****P < 0.0001 vs. basal. **E** Lysates were prepared from HEK293T cells incubated with rapamycin or torin1 (0.05 to 1 μM) for 24 h and endogenous immunoblotted as indicated, with quantitation presented as mean phosphorylation (fold vs. basal) ± SEM, *n* = 4. **F** iRAPWT or iRAPKO MEFs were incubated with 1 μM 4-OHT for 96 h and prepared lysates immunoblotted as indicated. Data presented as mean phosphorylation (a.u. arbitrary units) ± SEM, *n* = 3. Statistical analyses were performed by one-way ANOVA with Dunnett’s multiple-comparisons test. n.s. not significant vs. vehicle untreated. COS7 cells expressing GST fusions (GST-α1) of (**G**) α1β1γ1 or (**H**) α1β2γ1 were incubated with phenformin (2 mM, 1 h), H_2_O_2_ (5 mM, 45 min) or 2-deoxy-glucose (2DG; 25 mM, 30 min), and GST-purified AMPK was immunoblotted as indicated. **I** Lysates were prepared from immortalised MEFs (WT or SIN1^−/−^), incubated under conditions of serum starvation overnight ± subsequent serum re-addition (1 h), and immunoblotted as indicated. Representative immunoblots from three independent experiments are shown.

To examine mTORC1-regulation of β-pS182/184 in mammalian cells over a longer period, we treated HEK293T cells expressing various FLAG-tagged AMPK γ1-complexes with 250 nM torin1 for up to 24 h and analysed lysates by immunoblot and FLAG-immunoprecipitated AMPK by LC-MS. α1-pS347 (FLAG-immunoprecipitated to remove an interfering non-specific band detected in lysates by the antibody^32^), α2-pS345 and α2-pS377 in these γ1-complexes were tracked as markers for mTORC1 inhibition, with time-dependent reductions in phosphorylation mirroring those reported previously^33^ (Fig. 5A–D). Consistent with the shorter torin1 incubations (Fig. 3B–D), dephosphorylation of β2-pS184 and β1-pS182 occurred gradually over time in both α1- and α2-complexes and generally stabilised by 8 h (Fig. 5A–D). Lower β2-pS184 immunoreactive signals after this time point arose from loss of β2 content, presumably due to inhibition of protein synthesis. However, even at 24 h, ~50% (immunoblot) or 81-85% (LC-MS) of basal β-pS182/184 signals were retained in all complexes.

Analysing endogenous AMPK in HEK293T cells by immunoblot, 24 h incubations with up to 1 μM torin1 or rapamycin had no substantial effects on β1-S182 phosphorylation despite robustly reducing signals for AMPK α2-pS345 and 4E-BP1-pT37/46 (Fig. 5E). Phosphorylation of β2-S184 in the basal state was poorly detectable, despite β2-AMPK making up ~ 40% of total AMPK complexes in these cells, and it did not diminish after 24 h incubation with 1 μM rapamycin but was undetectable after 24 h incubation with 1 μM torin1. It is worth noting that, while the epitope for the total β antibody is fully conserved between β1 and β2 (surrounding His233), the β-pS182/184 phospho-antibody used in these experiments was raised against β1-pS182 and may have a weaker affinity for β2-pS184 due to sequence divergence NH_2_-terminal to the phosphosite.

To examine the effect of genetic loss of mTORC1 signalling on β1-pS182, we used an immortalised MEF cell line in which downregulation of raptor expression can be induced with 4-OHT (iRapKO)^56^. After a 96 h incubation with 4-OHT, we observed ~70% loss of raptor content in iRapKO MEFs which was accompanied by ~37% decrease in 4E-BP1-pT37/46 (Fig. 5F). No changes in raptor expression or phosphorylation of 4E-BP1-T37/46 were observed in the counterpart iRapWT cell line, and β-pS182 levels were unchanged in both cell lines after 4-OHT incubation.

Classical AMPK activators that disrupt mitochondrial ATP production (phenformin, H_2_O_2_) or glycolysis (2-deoxyglucose (2-DG)) and inhibit mTORC1 signalling had no bearing on β1-pS182 or β2-pS184 levels on AMPK expressed in COS7 cells (Fig. 5H, I). Overnight serum starvation and 1 h re-addition (mTORC1 inhibiting and activating, respectively) had no effect on endogenous β1-pS182 in MEFs, at least as detected by immunoblot (Fig. 5K). Because of their high levels of phosphorylation and low dephosphorylation kinetics, we considered that β-S182/184 might be co-translationally modified and subsequently internalised following protein folding. For example, mTORC2 is known to phosphorylate turn motif sites in AGC kinases like Akt during translation to ensure stability of the polypeptide upon ribosome release^57,58^. We investigated whether mTORC2 could also be an upstream kinase for β-S182/184, by taking advantage of immortalised MEF cells genetically devoid of the critical mTORC2 component SIN1 (SIN1^−/−^). SIN1^−/−^ MEFs are characterised by loss of phosphorylation of the mTORC2 substrate Akt-S473 under serum-replete conditions or following a serum starvation and replenishment cycle (Fig. 5J)^59^. As for WT MEFs, β1-pS182 levels were unchanged in SIN1^−/−^ MEFs following overnight serum starvation or subsequent 1 h serum replenishment (Fig. 5K). These results indicate that while β1-S182 and β2-S184 are likely direct substrates for mTORC1, mTOR-independent signalling pathways and/or low phosphatase accessibility also contribute to net phosphorylation of β1-S182, and probably β2-S184.

### Functional roles for β-S182/184 phosphorylation

**Fig. 6:**
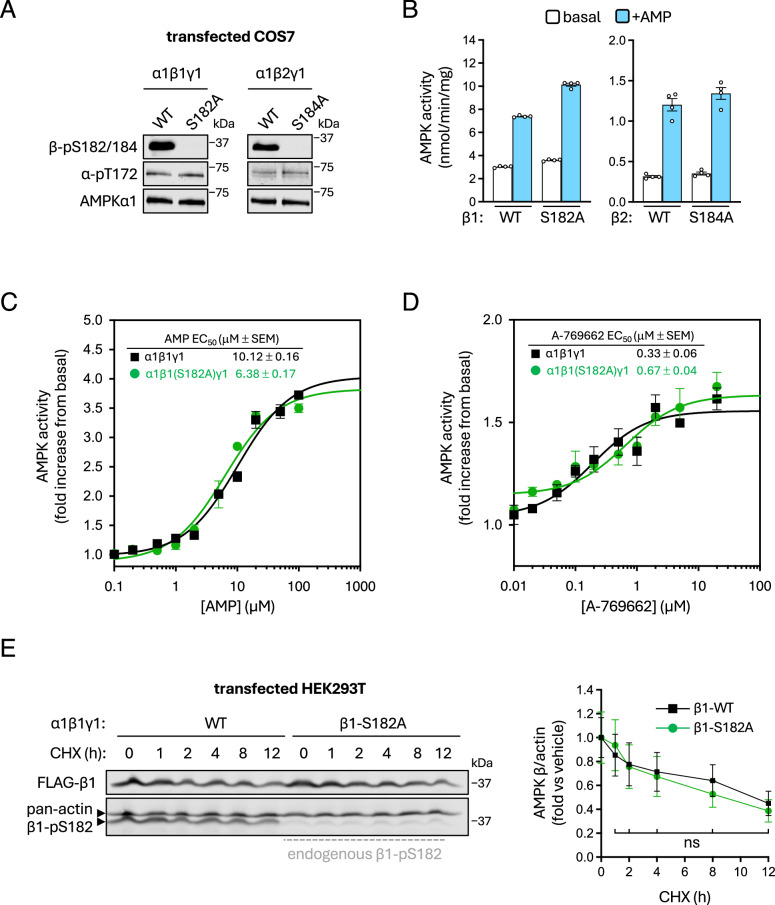
Investigating regulatory functions of β-pS182/184. **A** COS7 cells, transfected to express AMPK α1β1γ1 or α1β2γ1 (WT and β-S182/184 mutants), were grown in complete growth media. Prepared lysates were immunoblotted as indicated. **B** Sensitivities of AMPK α1β1γ1 (WT or β1-S182A mutant), FLAG-purified from COS7 cells incubated in complete growth media, to AMP (100 μM) were determined by radiolabelling of SAMS peptide substrate. Data presented as mean AMPK activity (nmol.min^−1^ mg^−1^) ± SEM, *n* = 4. Statistical analyses were performed by one-way ANOVA with Dunnett’s multiple-comparisons test. *P < 0.05, **P < 0.01, ***P < 0.001, ****P < 0.0001. Kinetics of FLAG-purified AMPK α1β1γ1 (WT or β1-S182A mutant) from COS7 cells in response to increasing doses of (**C**) AMP or (**D**) A-769662 were determined by radiolabelling of SAMS peptide substrate. Data presented as average fold change in AMPK activity (nmol min^−1^ mg^−1^) vs. basal ± SEM, *n* = 4. **E** Lysates were prepared from cycloheximide-treated (CHX; 1 μM for up to 12 h) HEK293T cells, expressing AMPK α1β1γ1 (WT or β1-S182A), and immunoblotted as indicated. Data presented as average fold change in β1/actin protein content vs. untreated ± SEM, *n* = 3. Statistical analyses were performed for WT vs. β1-S182A at each time point by unpaired *t* test. Representative immunoblots from three independent experiments are shown.

β1-S182 was originally identified as a near-stoichiometrically phosphorylated AMPK residue in mouse liver, with the β2 equivalent S184 site found to be partially phosphorylated in mouse skeletal muscle^51,52^. Subsequent functional analyses using non-phosphorylatable β1-S182A mutant AMPK extracted from transfected COS cells revealed this phosphosite did not influence basal AMPK activity or sensitivity to AMP^60^. We replicated and confirmed these findings for both β1-S182 and β2-S184 (Fig. 6A–C). β1-S182 lies adjacent to the αC-interacting helix (β residues 162–173) important for ADaM site drug regulation^16^, which led us to consider that phosphorylation may influence AMPK drug sensitivity. Using a radiometric kinase assay, EC_50_ values and maximal fold activation for the β1-specific ADaM site drug A-769662 were comparable between WT and β1-S182A mutant AMPK isolated from COS7 cells (Fig. 6D). Additionally, the half-lives of transfected, FLAG-tagged β1 WT and S182A mutant proteins were similar in HEK293T cells treated for 12 h with cycloheximide (CHX), an inhibitor of protein synthesis, demonstrating that β1-pS182 does not affect AMPK heterotrimer stability (Fig. 6E).

**Fig. 7:**
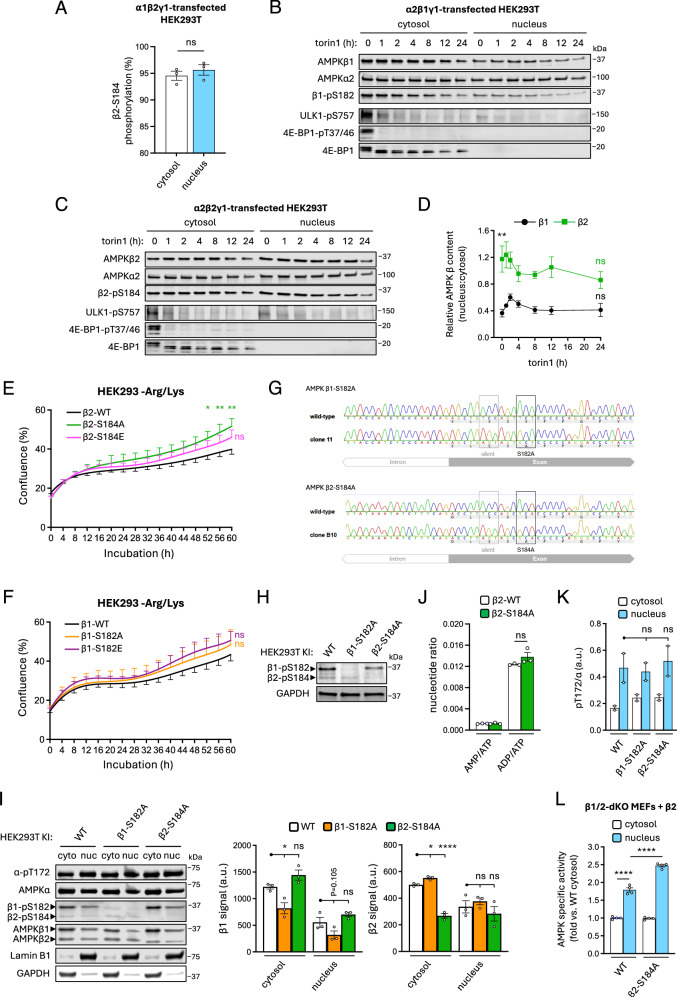
Dephosphorylation of β1-pS182 and β2-pS184 is not a prerequisite for nuclear AMPK transport but for β2 promotes cell proliferation under conditions of amino acid stress. Cytosolic and nuclear fractions were prepared from α1β2γ1-, α2β1γ1- or α2β2γ1-transfected HEK293T cells incubated in complete media. **A** Stoichiometries of β2-pS184 on FLAG-α1β2γ1 in cytosolic and nuclear fractions determined by LC-MS using peptide and phosphopeptide area under the curve. Data presented as mean % stoichiometry ± SEM, *n* = 3. Statistical analyses were performed by unpaired *t* test. GST-tagged (GST-α) (**B**) α2β1γ1- and (**C**) α2β2γ1-expressing HEK293T cells were incubated with torin1 (250 nM) for up to 24 h, cytosolic and nuclear fractions were immunoblotted as indicated. **D** Data from (**B**) and (**C**) are presented as relative AMPK β content (nucleus:cytosol ratio) ± SEM, *n* = 4. Statistical analyses were performed by unpaired *t* tests vs. basal (β1 vs. β2, **P < 0.01; 0 h vs. 24 h, ns not significant). Real-time proliferation analysis of HEK293 cells expressing FLAG-fusions of either (**E**) α2β2 (WT or β2-S184A/E mutants) or (**F**) α2β1 (WT or β1-S182A/E mutants), after switching to arginine- and lysine-free (−Arg/Lys) medium from complete growth medium. Data presented as mean % confluence ± SEM, *n* = 6. Statistical analyses at each time point were performed by 2-way ANOVA vs. respective WT. *P < 0.05, **P < 0.01. Validation of HEK293T β1-S182A and β2-S184A KI cell lines by (**G**) DNA sequencing, and (**H**) immunoblot. **I** Nuclear and cytosolic fractions were prepared from WT, β1-S182A KI and β2-S184A KI HEK293T cells and immunoblotted as indicated. Data presented as mean β1 or β2 signal (a.u. arbitrary units) ± SEM, *n* = 3. Statistical analyses were performed by one-way ANOVA with Dunnett’s multiple-comparisons test. *P < 0.05, ****P < 0.0001. **J** Adenine nucleotides were PCA-extracted from WT and β2-S184A KI HEK293T cells incubated in complete growth media and quantified by LC-MS/MS. AMP/ATP and ADP/ATP ratios are depicted here, ± SEM, *n* = 3. Statistical analyses were performed by one-way ANOVA with Dunnett’s multiple-comparisons test. n.s. not significant. **K** Data from (**I**) are presented as mean α-pT172/α (a.u. arbitrary units) ± SEM, *n* = 2. Statistical analyses were performed by one-way ANOVA with Dunnett’s multiple-comparisons test. n.s. not significant. **L** Cytosolic and nuclear fractions were prepared from β1/2-dKO immortalised MEFs expressing β2 WT or S184A mutant, and activities of FLAG-immunoprecipitated AMPK measured by radiolabelling of SAMS synthetic peptide. Data presented as fold change in mean AMPK specific activity vs. cytosolic WT ± SEM, *n* = 4. Statistical analyses were performed by 2-way ANOVA vs. WT nuclear activity. ****P < 0.0001. Representative immunoblots from three independent experiments are shown.

Singular expression of GFP-tagged, S182A-mutated β1 in HEK293T cells was reported to display a distinct ‘nuclear shift’ compared to WT β1-GFP, suggesting that loss of β1-S182 phosphorylation promotes nuclear translocation of AMPK^60^. We compared the basal stoichiometries of β2-pS184 in cytosolic and nuclear fractions of FLAG-α1β2γ1-transfected HEK293T cells by LC-MS and found this residue was near-maximally phosphorylated in both compartments, indicating that dephosphorylation of β2-pS184 is not a prerequisite for AMPK nuclear localisation (Fig. 7A). Torin1 (250 nM) incubation of HEK293T cells expressing GST-tagged α2β1γ1 or α2β2γ1 reduced β-pS182/184 signals by up to 50% after 24 h but had no long-term effect on relative nuclear:cytosol abundance of β1 after an initial small increase and did not cause a significant decrease in the proportion of nuclear β2 (Fig. 7B–D). Consistent with previous findings^61,62^, HEK293T cells expressing β2-AMPK had increased proportional abundance of nuclear AMPK versus those expressing β1, despite both β1 and β2 being co-expressed with α2 which contains a nuclear localisation signal^60^ (Fig. 7D). Analysis by live-cell imaging revealed that HEK293 cells transfected to express β2-S184A mutant displayed significantly enhanced proliferation compared to WT under conditions of amino acid starvation, whereas transfection with the phosphomimetic β2-S184E mutant produced an intermediate, non-significant effect. Transfection with β1-S182A or S182E mutants had no effect on cell proliferation compared to WT β1 (Fig. 7E, F).

To explore whether β-pS182/184 regulates nuclear translocation of endogenous AMPK, we used CRISPR-Cas9 gene editing to generate HEK293T cell lines harbouring constitutive Ala knock-in (KI) substitutions of β1-S182 or β2-S184 (Fig. 7G). Complete losses of β1-S182 or β2-S184 phosphorylation in the respective KIs were validated by immunoblot (Fig. 7H). Both of these cell models of chronic β-S182/184 dephosphorylation were characterised by reduced total expression of the respective β-isoform. Despite this, nuclear content of β2-AMPK in the β2-S184A KI was unchanged relative to WT (Fig. 7I). While this indicates a preference for β2-S184A AMPK to congregate in the nucleus in this cell model, the effect was not significant (WT vs β2-S184A KI, *P* = 0.161, data not shown). Neither KI affected basal adenine nucleotide ratios, as measured by LC-MS (Fig. 7J).

Notably, α-pT172 signals were 2-2.5-fold higher on nuclear AMPK relative to the cytosolic pool but did not vary significantly between cell lines (Fig. 7K). Our attempts to assay endogenous nuclear AMPK activity in WT and β2-S184A KI cells were confounded by insufficient recovery of material following antibody immunoprecipitation, therefore we expressed FLAG-tagged human AMPK β2 (WT or S184A) in β1/2-double knockout MEFs^20^ and assayed activities of nuclear and cytosolic AMPK following FLAG immunoprecipitation. After AMPK normalisation for total immunoprecipitated AMPK, specific activity of nuclear WT AMPK was elevated relative to cytosolic AMPK and was further increased in the nuclei of the β2-S184A-expressing cells (Fig. 7L). These findings confirm, and further demonstrate, that β-S182/184 phosphorylation does not overtly affect AMPK allosteric regulation or protein turnover, nor does it impede AMPK nuclear translocation, whereas loss of β2-S184 phosphorylation may increase specific activities of β2-AMPK found in the nucleus.

### Loss of β2-S184 phosphorylation modifies the expression of genes involved in glucose-handling and elevates Akt signalling

**Fig. 8:**
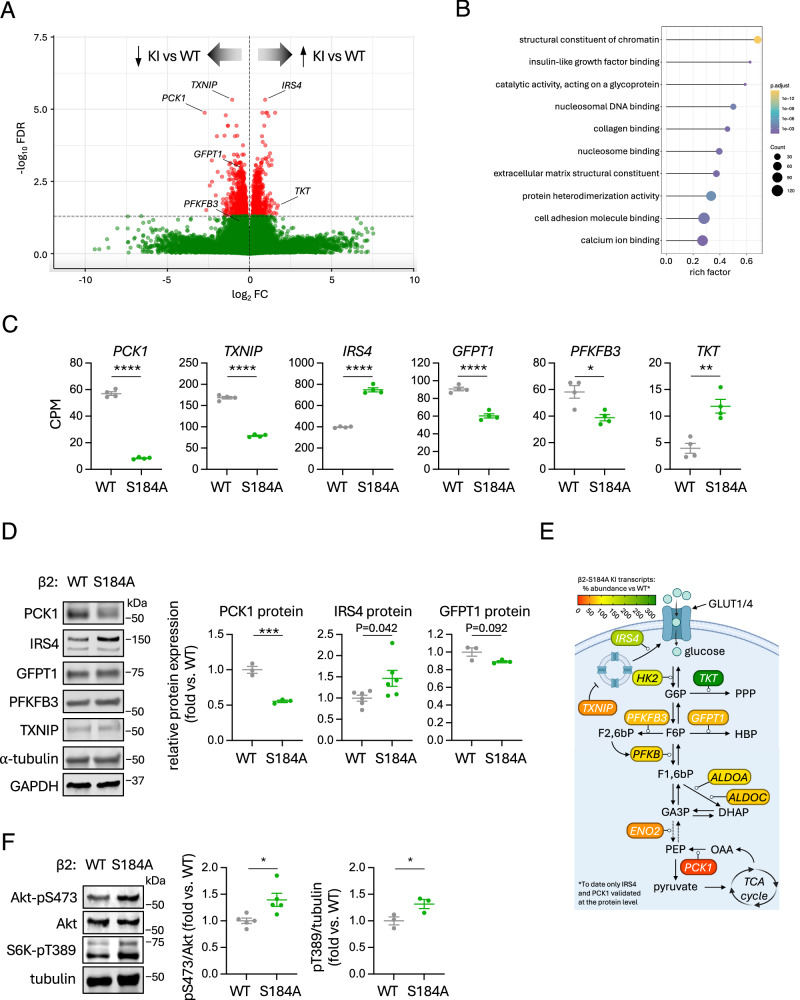
Analysis of metabolic rewiring using a cell model with chronic loss of β2-S184 phosphorylation. **A** Volcano plot representing the RNA-seq differential expression analysis of WT versus β2-S184A KI HEK293T cells. Plot was generated using an online tool provided by the Molecular and Genomics Informatics Core (MaGIC, Rutgers Health New Jersey Medical School; https://volcano.bioinformagic.tools/). **B** Enrichment analysis of genes differentially expressed between WT vs. S184A KI HEK293T cells. Graph shows the top 10 Gene Ontology (GO) terms with most genes annotated. Dot diameter indicates the number of differential genes; colour depth indicates significance. **C** Comparisons of differentially expressed genes involved in glucose uptake and handling, extracted from our RNAseq dataset. Data presented as mean CPM ± SEM, *n* = 4. Statistical analyses were performed by unpaired *t* test. *P < 0.05, **P < 0.01, ****P < 0.0001. **D** Immunoblot validation of data presented in (**C**). Lysates were prepared from WT and β2-S184A KI cells grown in complete media and immunoblotted as indicated. Data presented as fold change in mean expression vs. WT ± SEM, normalised to either GAPDH or tubulin, *n* = 3 (PCK1, GFPT1) or *n* = 6 (IRS4). Statistical analyses were performed by unpaired *t* test. ***P < 0.001. **E** Schematic showing genes with significantly altered expression in β2-S184A KI cells and the positions of their protein products in glucose metabolism pathways and shunts including GLUT1/4 translocation, glycolysis, pentose phosphate pathway (PPP) and hexose biosynthesis pathway (HBP). Proteins are coloured according to the mean relative % expression of their mRNA transcript in β2-S184A KI compared to WT, *n* = 4. **F** Lysates were prepared from WT and β2-S184A KI cells grown in complete media and immunoblotted for Akt-pS473 (*n* = 5) and S6K-pT389 (*n* = 3). Data presented as mean fold change in phosphorylation vs. WT ± SEM. *P < 0.05. Representative immunoblots from 3 independent experiments are shown.

To probe the functional ramifications of increased nuclear β2-AMPK activity induced by loss of β2-S184 phosphorylation, we performed comparative RNA-seq to assess changes in gene expression between WT and β2-S184A KI HEK293T cells. Of the total 41,446 transcripts detected, β2-S184A mutation induced significant (*P* < 0.05) up-regulation of 2958 and down-regulation of 3405 (Fig. 8A). Functional annotation of the significantly regulated genes showed an enrichment of genes involved in chromatin structure, insulin-like growth factor binding, collagen binding and nucleosome binding among the upregulated genes (Fig. 8B). Some of the most significant changes were seen in the transcription of key genes involved in gluconeogenesis (*PCK1*: 15% vs WT), glycolysis (*PFKFB3*: 69%), glucose uptake (*IRS4*: 193%, *TXNIP*: 48%), and the hexosamine biosynthesis pathway (HBP) (*GFPT1*: 68%) (Fig. 8C). Gene expression for the major pentose phosphate pathway (PPP) enzyme transketolase was also increased in β2-S182A KI cells (*TKT*: 332% vs WT), although detected abundance was generally low. We validated the RNA-seq dataset by immunoblotting for protein expression and observed 45% loss of PCK1 and 146% increase in IRS4 in the KI cells relative to WT (Fig. 8D). Changes in the levels of proteins arising from other altered mRNA species such as GFPT1, PFKFB3 and TXNIP were not significant, possibly indicating compensatory mechanisms affecting turnover. Figure 8E shows a schematic of the relative positions that protein products of these differentially expressed genes occupy in glucose uptake and metabolism pathways. IRS4 has been implicated in the progression of many cancers by maintaining pro-survival PI3K/Akt signalling^63^. Consistent with increased IRS4 protein expression in β2-S184A KI cells, activating phosphorylation of Akt-S473 was significantly elevated in these cells compared to WT even under growth conditions, as was phosphorylation of the mTORC1 substrate S6K-T389 (Fig. 8F).

## Discussion

Here we combine targeted LC-MS and conventional approaches to generate comprehensive phosphorylation profiles of all 12 human AMPK complexes expressed in mammalian cells and examine their fluctuations in response to mTORC1-inhibiting conditions. Our LC-MS approach, adapted from Steen et al.^38^, provides a new level of insight on AMPK complex-specific phosphosite stoichiometries without the need for expensive and time-consuming analysis using phosphosite-specific antibodies. However, we note several caveats to the method including currently being restricted to an over-expression system (necessitated to generate sufficient, isoform-defined material for quantification), expression of γ2 and γ3 isoforms that are not normally found at high levels in HEK293T cells, long LC-MS run times and the need to conduct all comparative analyses on a single LC-MS platform to preserve phospho- and dephospho-peptide flyability ratios. We were unable to detect α-pT172/174 (activating) and β1-pS108 (metabolite/drug sensitising) phosphosites by this method, although stoichiometries for these have been previously estimated using immunoblot techniques^10,11^. Nevertheless, under optimal cell growth conditions we demonstrate substantial heterogeneity in phosphorylation of α-T172/174, β-S108 and a range of α-subunit ST loop sites (some inhibitory) between complexes (Table 3). 13 of the 19 phosphorylation sites we detected by LC-MS have unknown function, including four in the γ2-NTE (S113, S143, S162 and S196) and the abundantly phosphorylated β-S182/184.

Our first key finding is that the γ2-NTE may represent a “hot-spot” for mTORC1 signalling, with each phosphosite we detected displaying sensitivity to pharmacological inhibition of mTOR. We consider this may well be an under-representation since we were unable to detect five other Ser-Pro sites in the γ2-NTE by LC-MS. Within the γ2-NTE, pS196 was detected in cells at phosphorylation stoichiometries similar to α2-S345 (~40–50%) and was rapidly dephosphorylated to low levels after 4 h exposure to torin1 (Fig. 3). γ2-S196 phosphorylation has previously been shown in separate high-throughput studies to be modulated by insulin in adipocytes and glucose availability in pancreatic β-cells^64,65^. The γ2-NTE is predicted to interact with the AMPK catalytic domain to regulate α-T172 phosphorylation^45^, raising the possibility that mTORC1 has a hand in controlling γ2-AMPK activity. Intriguingly, activating mutations in γ2-AMPK have been associated with increased mTORC1 signalling in the hypothalamus and cardiomyocytes, along with elevated rates of *mTOR* transcription in the pancreas^66,67^. Hyperactivation of both γ2-AMPK *and* mTORC1 trigger enrichment of genes that drive proliferation of pancreatic β-cells, a process normally antagonised by AMPK^66,68^, highlighting a more nuanced interplay between these two kinases. Given the AMPK γ2 isoform is a critical determinant of cardiac physiology, ghrelin-induced feeding behaviour in the hypothalamus and glucose-stimulated insulin secretion in the pancreas^66,69^, this underscores the importance of delineating the precise function of putative mTORC1 phosphosites on the γ2-NTE.

Our second key finding is that β1-S182 and β2-S184 are direct substrates of mTORC1 in vitro, confirming phosphoproteomics results from insulin-treated HEK293 cells^34^. Cellular phosphorylation at these sites was sensitive to torin1 but not chronic 70% loss of raptor expression, and even then dephosphorylation occurred slowly and incompletely (Fig. 5). These dynamics can be explained by a combination of low accessibility of these sites to phosphatases, sufficient residual mTORC1 activity in the iRapKO MEFs to maintain phosphorylation of “strong” substrates such as β-pS182/184 and 4E-BP1-pT37/46^55^, and the existence of alternate and compensatory β-S182/184 kinases. Interestingly, mTORC1-mediated phosphorylation of β1-S182/184 was heavily influenced by the regulatory α-ST loop, since replacement of this region in α1 for the α2 sequence intrinsically inhibited β-S182/184 phosphorylation by mTORC1 (Fig. 4). Whether phosphorylation of an α1 or α2 ST loop phosphosite(s) affects the ability of mTORC1 to access β-S182/184 is an exciting area for future investigation. It is worth highlighting that Akt, which in response to growth factors indirectly activates mTORC1 by relieving inhibition by TSC2^62^, is an α1 ST loop kinase and potential candidate for facilitating β-S182/184 phosphorylation. In agreement with previous analysis of the highly-expressed β2-subunit extracted from skeletal muscle^51,52^, α2β2γ2 and α2β2γ3 complexes had the lowest AMPK β-S182/184 phosphorylation stoichiometry, suggesting greater rates of turnover and a more prominent functional role. Our observations that dephosphorylation of β2-S184 preferentially upregulates nuclear AMPK activity, seemingly independently of changes to α-pT172, and enhances cell proliferation under nutrient restriction, suggests β2-pS184 plays a critical role in AMPK function.

All reported AMPK crystal structures are in the β-S182/184 dephosphorylated state. To our knowledge, only one active AMPK structure contains a resolved β-S182/184 residue (PDB: 4RER). Here, β2-S184 is found in the highly flexible β-linker loop connecting the β-CBM to the α-γ-SBS and occupies a position proximal to the activation loop, 13 Å from α1-T174^70^. It is possible that phospho-turnover at the β2-S184 site alters the conformation of the activation loop to control AMPK activity or substrate recruitment, yet why this stimulatory effect of dephosphorylated β2-S184 on AMPK activity is only apparent in the nucleus, is difficult to reconcile. We note that α-pT172 is higher in nuclear versus cytoplasmic extracts from HEK293T cells irrespective of the level of β2-S184 phosphorylation, which is either because of greater colocalization with an α-T172 kinase, or nuclear AMPK adopts a conformation that protects this site against phosphatase pressure. Furthermore, the rate at which β2-pS184 is dephosphorylated in vitro is not determined by starting levels of α-pT172. Thus, the functional significance of β2-S184 being structurally modelled next to the activation loop and α-T172 remains unclear. In addition to the well-characterised Rag-GTPase-dependent scaffolding of mTORC1 to the lysosome for activation^24–27^, distinct modes of mTORC1 activation in the nucleus^71^ lend credence to the idea that β2-pS184 dephosphorylation-phosphorylation cycles could similarly occur in the nucleus. In that regard, nuclear AMPK may encounter a unique pool of phosphatases that regulate a range of AMPK phosphosites, which combined with loss of β2-pS184, elevates its activity above the cytosolic fraction. As such, there may exist a nuclear β2-pS184 phosphatase(s) mobilised under cellular stress conditions like nutrient deprivation. As mentioned, β2-S184 dephosphorylation enhances cell proliferation during nutrient stress, and it is noteworthy that dephosphorylation of the mTORC1 substrate on AMPK α2-S345 has the exact opposite effect on cell proliferation under identical conditions^32^. mTORC1 inhibition and α2-S345 dephosphorylation triggers lysosomal targeting of AMPK and downstream signalling in the cytoplasm (e.g., enhanced ACC phosphorylation)^33^. Therefore, compartment-specific modes of mTORC1 and AMPK signalling likely exist to fine-tune prevailing cellular responses to changes in nutrient availability. This is ultimately made possible by the existence of up to 12 unique AMPK heterotrimers, each differentially regulated by phosphorylation.

Our third key finding, that acute β2-S184 dephosphorylation sustains cell growth during nutrient limitation may explain, albeit in part, the complex role AMPK plays in cancer^72^, whereby in the established tumour AMPK endows cancerous cells with an ability to proliferate despite their poorly vascularised and nutrient-deprived microenvironment. Indeed, close inspection of the TCGA Pan-Cancer Atlas^73^ demonstrates that the gene encoding β2 (*PRKAB2*) is amplified in a range of cancers, including breast, liver and lung. It is conceivable that changes in the expression of genes (which we identified with RNA-seq analysis) and their protein products implicated in glucose metabolism contribute to the superior rate of proliferation in cells with higher nuclear AMPK activity. Chronic loss of β2-S184 phosphorylation enhanced basal Akt/mTORC1 signalling in HEK293T cells, a major oncogenic signalling pathway, possibly a result of elevated expression of the insulin receptor substrate IRS4 (Fig. 8D). High expression of IRS4 in mammalian cells has been linked to upregulated Akt/mTORC1 signalling, whereby loss of IRS4 stunted cellular growth^74^. These results suggest AMPK transcriptionally regulates its own phosphorylation by mTORC1 and that both kinases can be active concomitantly, likely in separate cellular compartments. We also observed a substantial reduction in the expression of PCK1 at both the gene and protein level. PCK1 is the rate-limiting enzyme in gluconeogenesis and Akt has notably been shown to phosphorylate PCK1 to drive non-canonical functions that upregulate lipogenesis and cancer cell proliferation^75^. Compensatory activation of PCK1 by Akt in cells devoid of β2-S184 phosphorylation is one possibility and is an area for further study, as is examination of AMPK nuclear substrates (e.g., transcription factors) under conditions of low β2-S184 phosphorylation. When considering the two top-ranked pathways induced by cells expressing constitutively dephosphorylated β2-S184 are “structural constituent of chromatin” and “insulin-like growth factor binding”, we speculate nuclear AMPK engages with a cluster of substrates (either specific to AMPK in the β2-S184 dephosphorylated state or generally arising through increased AMPK nuclear activity) that collectively induce the pro-growth phenotype observed in human cells (Fig. 7E). It is worth mentioning that some downstream nuclear targets of AMPK are implicated in tumourigenesis. For example, AMPK-mediated activation of TFEB, a master transcriptional regulator of autophagosome and lysosome biogenesis, has been shown to promote chemoresistance^76^. Our results also imply that chronic β2-pS184 dephosphorylation in cancer cells (perhaps arising from sustained periods of low mTORC1 activity) contributes to survival and growth by providing a safeguard mechanism to preserve mTORC1 signalling through IRS4/Akt. Clinical effectiveness of anti-cancer mTOR inhibitors may therefore be improved by combinatorial therapy involving a β2-AMPK-inhibiting agent, such as compound MT47-100 we described previously^77^.

In skeletal muscle cells, simply exchanging β2 with β1 in complex with α2 (that contains a nuclear localisation signal) renders AMPK refractory to nuclear translocation^61^, while in rat liver, α1-AMPK translocates to the nucleus in a circadian-dependent manner commensurate with peak expression of β2 and not β1^78^. The myristoylation-deficient β1-G2A mutant, that abolishes membrane-bound organelle (i.e., lysosome) association, can however translocate to the nucleus upon stimulation^61^. Whilst we were unable to confirm a previous report showing loss of β-S182/184 phosphorylation strongly promotes AMPK nuclear abundance^60^, overall it appears that the intrinsic properties of the β2 isoform are critical determinants of nuclear AMPK biology. Like β2, the gene encoding α1 (*PRKAA1*) is often amplified in cancer which has led to a model in which this AMPK isoform serves as a tumour promoter in some contexts^72^. A recent investigation demonstrated that caspase-3 cleavage of the extreme COOH-terminal portion of the α1 isoform removes a nuclear export signal and sequesters AMPK in the nucleus, with nuclear AMPK enhancing cell survival in response to genotoxic stress^79^. These α1-containing complexes are thought to be activated by CaMKK2 by intranuclear Ca^2+^ flux^80^, thereby establishing a link between nuclear AMPK and resistance to chemotherapeutic DNA-damaging agents. It is now essential to determine whether the propensity for α1β2-containing AMPK to infiltrate the nucleus accounts for their oncogenic properties in certain cancers, and pinpoint the mechanisms responsible for their activation.

## Methods

### Materials

Rapamycin (R8781), AMP (A1752), ATP (A2383), phenformin (P7045), 2-deoxy-D-glucose (2DG; D6134), H_2_O_2_ (18304), cycloheximide (C7698) and 4-hydroxytamoxifen (4-OHT; H6278) were from Sigma-Aldrich. Torin1 (S2827) was from Selleckchem and A-769662 (ab120335) was from Abcam. All restriction enzymes used for cloning were from New England Biolabs (NEB).

### Plasmid constructs and mutagenesis

All primers used for cloning were ordered from Sigma-Aldrich (Table 4). All new constructs were confirmed by Sanger sequencing (Micromon, Monash University, Australia). Residues are numbered according to mRNA accession AAA64850 (human α1, referred to as the short form due to differential Met initiation) and Uniprot ID P45646 (human α2). For bacterial expression of AMPK heterotrimeric chimera α2(α1ST), human DNA sequence α2_1-474_/α1_471-530_/α2_533-552_ was generated by Gene Universal (Newark, Delaware, United States) and cloned into the pET DUET-1 multiple cloning site (MCS) 1 (MCS2 already containing AMPK γ1) using MfeI/XhoI restriction sites in-frame with an NH_2_-terminal 6xHis tag^10^. Plasmid constructs containing human α2(α1Cterm)/β1 (his-α2_1-254_/α1_257-550_ in pET15b) and α2(α1RIM) (α2_1-347_/α1_350-392_/α2_397-552_ in pCMV) were kind gifts from Kei Sakamoto (University of Copenhagen). α2(α1RIM) was cloned into pET DUET-1 as for α2(α1ST). For bacterial expression of lambda phosphatase, DNA was cloned into pGEX-6P-2 using BamHI/NotI restriction sites in-frame with an NH_2_-terminal GST tag and PreScission protease site.

**Table 4:** Primers used for cloning

Construct (plasmid)	Type	Primer sequences
_6xHis_α2(α1ST) & _6xHis_α2(α1RIM) (pET DUET-1 MCS1)	Forward Reverse	5′ GGTAGGATCC**GCTGAGAAGCAGAAGCACGACG** 3′ 5′ GCATGCGGCCGC**TCAACGGGCTAA** **AGTAGTAATCAGACTGG** 3′
α2_no tag_ (α1ST) (pcDNA3.1(−))	Forward Reverse	5′ GGTACTCGAG**ATGGCTGAGAAGCAGAAGC** 3′ 5′ GCATAAGCTT**TCAACGGGCTAAAGTAG** 3′
γ1_FLAG_ (pcDNA3.1(−))	Forward Reverse	5′ GCATGCGGCCGC**ATGGAGACGGTCATTTCTTCA** 3′ 5′GCATGAATTCTCACTTGTCGTCATCGTCTTT GTAGTCTCCACC**GGGCTTCTTCTCTCCACC** 3′
γ2_FLAG_ (pcDNA3.1(−))	Forward Reverse	5′ GCATGCGGCCGC**ATGGGAAGCGCGGTTAT** 3′ 5′ GCATGAATTCTCACTTGTCGTCATCGTCTTT GTAGTCTCCACC**CTCCGTTTCTGTCTCCT** 3′
Lambda phosphatase	Forward Reverse	5′ GCATGGATCC**CGGTACTACGAAAAAATCGACG** 3′ 5′ GCATGCGGCCGC**TTAGGCACCTTCGCCCTGAACC** 3′

Complementary sequences are in bold.

For mammalian cell expression, AMPK α2/α1 chimera DNA was cloned into pcDNA3.1(−) using XhoI/HindIII restriction sites. Human AMPK γ1 and γ2 DNA sequences were cloned into pcDNA3.1(−) using NotI/EcoRI restriction sites. Human AMPK γ3 DNA sequence was generated and cloned into pcDNA3.1(−) using NotI/EcoRI restriction sites by Gene Universal. Mouse PP2C phosphatase was cloned into pcDNA3.1(−) using XbaI/HindIII restriction sites. All other plasmids used in this study have been described previously^10,19,20,81–83^.

For mutagenesis, primers containing the desired mutation, as detailed in Table 5, were ordered from Sigma-Aldrich. Both forward and reverse mutagenic primers were mixed with 10x PfuUltra buffer (NEB), DNA template (40 ng), dNTPs (Sigma-Aldrich), PfuUltra DNA polymerase (NEB) and nuclease-free H_2_O to a final volume of 50 μL. The DNA was amplified by PCR using a thermocycler (Bio-Rad). Dpn1 (NEB) was added to the reaction and incubated for 2 h at 37 °C to digest methylated parental DNA. The amplified DNA was transformed into α-select competent cells (Bioline) by heat shock and plated on Luria-Bertani broth (LB) agar plates containing 100 µg mL^−1^ antibiotics. Individual colonies were inoculated in 5 mL LB containing 100 µg mL^−1^ antibiotics and incubated overnight at 37 °C. Plasmid DNA was isolated using a Wizard Plus SV minipreps DNA purification kit (Promega) as per the manufacturers protocol.

**Table 5:** Primers used for site-directed mutagenesis

Mutation	Type	Primer sequences
β1-S182A	Forward Reverse	5′ TGAGCTGTCCAGT**GCT**CCCCCAGGACCCTA 3′ 5′ TAGGGTCCTGGGGG**AGC**ACTGGACAGCTCA 3′
β2-S184A	Forward Reverse	5′ CCTTTCCAGC**GCA**CCCCCAGGGC 3′ 5′ GCCCTGGGGG**TGC**GCTGGAAAGG 3′

Codon changes are in bold.

### Mammalian cell lines

Generation of β1/β2 double knockout (β-dKO) immortalised MEFs has been described previously^20^. iRapWT and iRapKO immortalised MEFs were a gift from Michael Hall (University of Basel). SIN1 knockout (SIN1^−/−^) immortalised MEFs were a gift from Bing Su (Shanghai Jiao Tong University School of Medicine). HEK293T/17, HEK293T, HEK293 and COS7 cells were from ATCC. All cell lines were maintained in Dulbecco’s Modified Eagle’s medium (DMEM; Sigma-Aldrich, D5796) supplemented with 10% fetal bovine serum (FBS; Assay Matrix) at 37 °C and 5% CO_2_.

### HEK293T β1-S182A and β2-S184A CRISPR knock-ins

Plasmids encoding epegRNAs for introduction of β1-S182A (RTT/PBS: 5′-AGAGCTGAGTAGT**G**/CTCCCCCAGGAC-3′; intended edit TCT>**G**CT) and β2-S184A (RTT/PBS: 5′-TCAGACCTTAGTAGC**G**/CACCCCCAGGGC-3′; intended edit TCT>**G**CA) under control of a human U6 promotor in pUC57 Kan were ordered from Gene Universal. To allow sorting of transfected cells by flow cytometry, pCMV PE2-T2A-Tomato was cloned from pCMV PE2 (Addgene #132775) through insertion of a T2A-tomato sequence after partial digest with AgeI and complete digest with EcoRI. HEK293T cells were seeded into a 12 well plate (200,000 cells/well) and after 24 h were transfected with 400 ng pCMV PE2-T2A-Tomato and 100 ng pUC57 pegRNA plasmid in 50 µL OptiMEM and 1.5 µL FuGene transfection reagent. Two days post-transfection, single TdTomato positive cells were sorted into 96 well plates and individual clones screened for presence of β1-S182A or β2-S184A and absence of wild-type sequence or unintended edits by Sanger sequencing (β1 forward primer: 5′-CACTCTTGGAACCAGTGCATC-3′; β2 forward primer: 5′-CTGTAGATCCCACAGGTCAC-3′) and immunoblot.

### Mammalian cell experiments and protein purification

For transient AMPK expression in HEK293T/17, HEK293T and COS7 cells and transient PP2c phosphatase expression in HEK293T cells, adherent cultures at 40–50% confluency were either triply transfected with full-length α1 or α2 (COOH-terminal FLAG fusion or untagged, pcDNA3.1 vector, or NH_2_-terminal GST fusion, pDEST27 vector), β1 or β2 (COOH-terminal MYC or FLAG fusion, pcDNA3.1 vector), and γ1, γ2 or γ3 (NH_2_-terminal HA fusion, pMT2 vector or COOH-terminal FLAG fusion, pcDNA3.1 vector), or singularly transfected with PP2C phosphatase (COOH-terminal FLAG fusion, pcDNA3.1 vector) in the presence of FuGENE HD transfection reagent (Promega) for 48 h as per the manufacturers protocol.

For expression of heterotrimeric AMPK in β-dKO immortalised MEFs, β1 or β2 (COOH-terminal FLAG fusion, LeGO-iG2 vector; WT and indicated mutants) were reintroduced by lentiviral transduction using the LeGO-iG2 system. The lentivirus-containing media was replaced with fresh media after 24 h and experiments were performed within 72 h of transduction. All cultures were incubated with fresh DMEM containing 10% FBS for 2 h prior to drug treatments.

To harvest cellular material, cells were first gently washed in ice-cold phosphate-buffered saline (PBS; Sigma-Aldrich) then scraped in ice-cold lysis buffer. For collection of whole cell material, cells were lysed in buffer containing 50 mM Tris pH 7.5, 150 mM NaCl, 10% (v/v) glycerol, 50 mM NaF, 5 mM sodium pyrophosphate, 1 mM EDTA, 1 mM EGTA, 1% (v/v) Triton X-100 and cOmplete protease inhibitor cocktail (Roche). Cell lysates were clarified by centrifugation (16,000 *g*, 3 min, 4 °C), flash-frozen in liquid-N_2_ and stored at −80 °C until subsequent analysis or processing. To purify FLAG-AMPK, complexes were immobilised by incubation of cell lysates with FLAG-M2 agarose (Sigma-Aldrich A2220) for 2 h at 4 °C. Following gentle centrifugation (1000 *g*, 3 min, 4 °C), the resin was washed twice in high salt purification buffer (50 mM HEPES pH 7.4, 1 M NaCl, 10% (v/v) glycerol, 0.02% (v/v) tween-20) then twice more in the same buffer with a lower salt concentration (150 mM NaCl; low salt purification buffer). To phosphorylate AMPK during purification, immobilised FLAG-AMPK complexes were incubated in the presence of 0.5 mM ATP, 2.5 mM MgCl_2_ (Merck Millipore), low salt purification buffer, 1 mM 1,4-dithiothreitol (DTT) and purified CaMKK2.1_156-588_-6xHis (10 µg for 2 × 10 cm plates of lysate) for 1 h at 22 °C with gentle rolling. The phosphorylation reaction was terminated by washing the reaction buffer out of the FLAG agarose with low salt purification buffer. To elute AMPK complexes, immobilised FLAG-AMPK was resuspended in FLAG elution buffer (low salt purification buffer supplemented with 1 mM DTT and 1 mg mL^−1^ FLAG peptide) and rotated overnight at 4 °C. FLAG purified AMPK was quantified by immunoblot using a highly pure bacterial-expressed AMPK complex as a standard. FLAG-PP2C phosphatase was purified using a similar protocol to untreated AMPK, with activity confirmed by α-pT172 immunoblot after incubating with a CaMKK2-treated AMPK substrate.

For separation of cytosolic and nuclear compartments, cells were harvested in cytosolic lysis buffer (10 mM HEPES pH 7.9, 10 mM KCl, 10% (v/v) glycerol, 50 mM NaF, 5 mM sodium pyrophosphate, 0.1 mM EDTA, 1.5 mM MgCl_2_, 0.5 mM DTT, 0.5% (v/v) NP40). Lysates were incubated on ice for 10 min, centrifuged (5000 *g*, 10 min, 4 °C) with the supernatant removed as the fraction containing cytosolic proteins. The pellet was then washed twice in cytosolic lysis buffer and pelleted by centrifugation (5000 *g*, 10 min, 4 °C) followed by resuspension in nuclear lysis buffer (20 mM HEPES pH 7.9, 400 mM NaCl, 10% (v/v) glycerol, 50 mM NaF, 5 mM sodium pyrophosphate, 1 mM EDTA, 1.5 mM MgCl_2_, 0.5 mM DTT), mechanically lysed in a Dounce glass homogeniser and incubated on ice for 30 min. The nuclear protein-containing fraction was clarified by centrifugation (16,000 *g*, 3 min, 4 °C). Protein content of all samples was determined by BCA assay (Pierce). All samples were flash-frozen in liquid N_2_ and stored at −80 °C until analysis. All cell lysis buffers were supplemented with a complete protease inhibitor cocktail (Roche).

### Bacterial protein expression and purification

Recombinant full-length heterotrimeric (NH_2_-terminal 6xHis-tagged) α1β1γ1, α1β2γ1, α2β1γ1, α2β2γ1 and chimeras α2(α1Cterm)β1γ1, α2(α1ST)β1γ1 and α2(α1RIM)β1γ1 AMPK were expressed in *E. coli* Rosetta 2 (DE3) (Merck Millipore) after double-transformation of pET Duet-1 (α- and γ1-subunits) and pCOLA (β1-subunit) plasmids (for WT and α2(α1ST) and α2(α1RIM) chimeras), or pET15b (α- and β1-subunits) and pCOLA (γ1-subunit) plasmids (for α2(α1Cterm) chimera), and purified as described previously for the _6xHis_α2β1γ1 complex^84^. In brief, expression cultures were grown at 37 °C to an optical density (OD_600_) of 3.0 before induction with 500 µM isopropyl-β-d-1-thiogalactopyranoside (IPTG; Gold Biotechnology) and incubation overnight at 16 °C. Cell pellets were resuspended in lysis buffer (50 mM Tris pH 7.6, 500 mM NaCl, 5% (v/v) glycerol, 50 mM imidazole, 2 mM β-mercaptoethanol (BME), 0.01 mM leupeptin, 0.1 mM AEBSF, 0.5 mM benzamidine hydrochloride), lysed using a precooled EmulsiFlex-C5 homogeniser (Avestin) and clarified via centrifugation (18,000 *g*, 30 min, 4 °C). Protein was bound to a HisTrap HP 5 mL Ni^2+^ column (Cytiva) at 1 mL min^−1^, washed with 10 column volumes of chilled Ni^2+^ column buffer (50 mM Tris pH 7.5, 500 mM NaCl, 5% (v/v) glycerol, 40 mM imidazole, 2 mM BME) and eluted with Ni^2+^ column buffer supplemented with 400 mM imidazole. Proteinaceous fractions were separated on a HiLoad 16/600 Superdex 200 gel filtration column (Cytiva) pre-equilibrated with AMPK size exclusion column buffer (AMPK SEC buffer; 50 mM Tris pH 8.0, 150 mM NaCl, 2 mM tris(2-carboxyethyl)phosphine (TCEP)). AMPK-containing fractions were pooled and concentrated to ~2 mg mL^−1^, flash-frozen in liquid-N_2_ and stored at -80 °C.

Recombinant lambda phosphatase was expressed in *E. coli* Rosetta 2 (DE3). Expression cultures were grown at 37 °C to an OD_600_ of 0.6 before induction with 500 µM IPTG and incubation overnight at 16 °C. Cell pellets were resuspended in lysis buffer (PBS supplemented with 1 mM EDTA, 2 mM BME, 0.01 mM leupeptin, 0.1 mM AEBSF, 0.5 mM benzamidine hydrochloride), lysed using a precooled EmulsiFlex-C5 homogeniser and clarified via centrifugation (18,000 *g*, 30 min, 4 °C). Protein was bound to glutathione sepharose 4B (GSH4B) resin (Cytiva) for 2 h at 4 °C, washed with 10 column volumes of chilled GSH4B column buffer (PBS supplemented with 1 mM EDTA, 2 mM BME) and eluted with GS4B column buffer being supplemented with 20 mM L-glutathione reduced (Sigma-Aldrich G4251). Proteinaceous fractions were separated on a HiLoad 16/600 Superdex 200 gel filtration column pre-equilibrated with lambda phosphatase SEC buffer (50 mM Tris pH 7.5, 150 mM NaCl, 2 mM TCEP). Lambda phosphatase-containing fractions were pooled and concentrated to ~6 mg mL^−1^, flash-frozen in L-N_2_ and stored at −80 °C. Recombinant CaMKK2.1_156-588_-6xHis was produced as described previously^13^. All purified protein was quality controlled by TOF-MS and SDS-PAGE Coomassie staining.

### Immunoblotting

Protein samples were separated on gradient SDS-PAGE gels (Bio-Rad) and transferred to an Immobilon-FL PVDF membrane (Merck Millipore). For nuclear and cytosolic fractions, 20 μg total protein was loaded per lane. After blocking the membrane with 2% non-fat dry milk dissolved in PBS + 0.1% (v/v) tween-20 (PBST; Sigma-Aldrich), membranes were incubated with primary antibodies for either 2 h at room temperature or overnight at 4 °C, as indicated in Table 6. Following repeated washes with PBST, fluorescently labelled secondary antibodies diluted in PBST were added as detailed in Table 6. The membranes were then washed extensively in PBST and visualised on the Odyssey Infrared Imaging System (LI-COR Biosciences) with immunoreactive bands analysed and quantified using Image Studio Software (LI-COR Biosciences).

**Table 6:** Antibodies used for immunoblotting

Antibody	Type	Source	Supplier	Cat#
AMPKα	Primary	Mouse	CST	2793
AMPKα-pT174/172	Primary	Rabbit	CST	2535
AMPKα1-pS347	Primary	Rabbit	^32^	n/a
AMPKα2-pS345	Primary	Rabbit	Abcam	ab129081
AMPKα-pS487/491	Primary	Rabbit	CST	2185
AMPKβ	Primary	Rabbit	CST	4150
AMPKβ1-pS108	Primary	Rabbit	CST	4181
AMPKβ1-pS182/184	Primary	Rabbit	CST	4186
FLAG tag	Primary	Mouse	CST	9146
FLAG tag	Primary	Rabbit	CST	14793
MYC tag	Primary	Mouse	CST	2276
ULK1	Primary	Rabbit	CST	4773
ULK1-pS757	Primary	Rabbit	CST	6888
4E-BP1-pT37/46	Primary	Rabbit	CST	9459
4E-BP1	Primary	Rabbit	CST	9452
Akt	Primary	Mouse	CST	2920
Akt-pS473	Primary	Rabbit	CST	9271
PCK1	Primary	Rabbit	CST	12940
IRS4	Primary	Rabbit	Abcam	ab52622
GFPT1	Primary	Rabbit	CST	5322
β-actin	Primary	Rabbit	CST	4970
α-tubulin	Primary	Mouse	CST	3873
Lamin B1	Primary	Rabbit	CST	13435
GAPDH	Primary	Mouse	Proteintech	60004-1-Ig
IRDye 680RD	Secondary	Mouse	LI-COR Biosciences	926-68070
IRDye 680RD	Secondary	Rabbit	LI-COR Biosciences	926-68071
IRDye 800CW	Secondary	Mouse	LI-COR Biosciences	926-32210
IRDye 800CW	Secondary	Rabbit	LI-COR Biosciences	926-32211

### Radioactive kinase assays

AMPK activity was determined as previously described^10^. Briefly, in a 25 µL reaction volume containing 100 µM SAMS peptide (Purar Chemicals, sequence: NH_2_-HMRSAMSGLHLVKRR-COOH), 5 mM MgCl_2_, 200 µM ATP, [γ-^32^P-ATP (Perkin Elmer), assay buffer (50 mM HEPES pH 7.4, 1 mM DTT and 0.02% (v/v) tween-20) and purified AMPK, phosphotransferase activity was conducted at 30 °C for 10 min and reactions were quenched by spotting 15 µL onto phosphocellulose ion-exchange chromatography paper (SVI Phosphocellulose, prepared in-house). The papers were repeatedly washed in 1% H_3_PO_4_ (Merck Millipore), added to vials containing 5 mL Ultima Gold liquid scintillation fluid (Perkin Elmer), and the level of ^32^P-transfer to the SAMS peptide was determined using a Tri-Carb 4810TR liquid scintillation counter (Perkin Elmer).

### In vitro phosphorylation reactions

Phosphorylation reactions were conducted at 30 °C by incubating bacterially-expressed AMPK substrate, 2.5 mM MgCl_2_, 500 µM ATP, AMPK SEC buffer, and the mTORC1 complex (Sigma-Aldrich; SRP0364) at a 1:10 kinase:substrate mass ratio for the duration indicated in the figure legends. Reactions were quenched by the addition of 1 µL of 500 mM EDTA. The quenched reaction mixture was either digested with trypsin for LC-MS/MS or diluted with Laemmli sample buffer and 100 ng of substrate analysed by immunoblotting.

### In vitro dephosphorylation reactions

Lambda phosphatase dephosphorylation reactions were conducted at 30 °C by incubating mammalian-expressed AMPK substrate, lambda phosphatase reaction buffer (50 mM HEPES pH 7.4, 100 mM NaCl, 500 µM MnCl_2_, 2 mM DTT, 0.01% (v/v) Brij-35), and bacterially-expressed lambda phosphatase as indicated in figure legend. PP2C dephosphorylation reactions were conducted by incubating 100 ng of mammalian-expressed AMPK substrate, PP2C reaction buffer (50 mM HEPES pH 7.4, 100 mM NaCl, 2 mM MgCl_2_, 2 mM DTT, 0.01% (v/v) Brij-35), and 100 ng of mammalian-expressed PP2C for 30 mins at 30 °C. Reactions were quenched by the addition of Laemmli sample buffer, and 100 ng of substrate was analysed by immunoblotting.

### LC-MS/MS analysis of peptides

All peptide analyses were carried out on a TripleTOF 5600 mass spectrometer (Sciex) operated with the turbo V DuoSpray ion source linked to an Ultimate 3000 RSLCnano system loading pump (Dionex) and Ultimate 3000 RS autosampler (Dionex). The LC-MS was operated using the Analyst TF v1.7.1 software (Sciex). Source and collision gas was provided by a Genius NM3G nitrogen gas generator (PEAK Scientific).

To prepare FLAG-immobilised AMPK for peptide analysis, protein was precipitated by the addition of 800 μL 100% methanol to the resin (>10x resin volume) and incubated on ice for 30 mins. The sample was centrifuged (12,000 *g*, 10 min, 4 °C), the supernatant discarded, and the resin dried under N_2_ gas. The dried resin was resuspended with 100 μL 50 mM Tris pH 7.5 (2x resin volume) and 200 ng of trypsin was added (Promega; made up to 100 ng µL^−1^ in H_2_O), after which samples were digested overnight at 37 °C with shaking to keep the resin suspended. Digests were quenched by addition of 1 µL of 5% formic acid (FA), resin pelleted by centrifugation (12,000 *g*, 10 min), before the supernatant was removed for LC-MS/MS analysis.

Digested AMPK was resolved on a Waters Acquity BEH peptide C18 column (100 mm×2.1 mm, 1.7 µm, 130 Å). The LC solvent system comprised of H_2_O with 0.1% FA for channel A, and 90% acetonitrile/10% H_2_O with 0.1% FA for channel B. A flow rate of 125 µL min^−1^ was used throughout a gradient program consisting of 2.5% B (2.5 min), 2.5 to 60% B (67.5 min), 60 to 100% B (20 min), 100% B (10 min), 100 to 2.5% B (2 min), 2.5% B (8 min). The mass spectrometer was set to time of flight-mass spectrometry (TOF-MS) acquisition mode and operated in positive ion mode with a mass range of 200–3000 *m/z* and accumulation time of 0.5 sec. Spray voltage was set to 5500 V, source temperature set to 300 °C, ion source gas 1 and 2 set at 35, curtain gas set at 15, and declustering potential set at 150 V.

LC-MS data was visualised, and peptides validated, using PeakView v2.2 software (Sciex) and peptide peak areas quantified using Skyline v21.1 (MacCross Lab Software). A list of AMPK peptides and charge states used for quantification is shown in Table 2. Phosphorylation stoichiometries were calculated from cognate phosphorylated (I_pP_) and dephosphorylated (I_P_) extracted ion count (EIC) peptide peak areas using Eq. (1) as described previously^39^. Charge states used for the quantification of each peptide are shown in Table 2.

1 $${\rm{Stoichiometry}}\,( \% )=\frac{k\cdot \left[{{\rm{I}}}_{{\rm{pP}}}\right]}{k\cdot \left[{{\rm{I}}}_{{\rm{pP}}}\right]\,+\,\left[{{\rm{I}}}_{{\rm{P}}}\right]}{{\times }}100$$

To normalise for differences in ionisation and detection efficiencies of the cognate I_pP_ and I_P_ on the MS, flyability ratios (*k*) were calculated and incorporated into Eq. (1). *k* was calculated using Eq. (2) from two samples of identical peptide concentration: one with low (**A**) and one with high (**B**) phosphorylation levels. To achieve this for all detected AMPK phosphosites, tryptic peptides were generated for α1β1γ2 and α2β2γ3 as described above (expressed in HEK293T cells under full growth conditions), except instead of quenching the reaction with FA, trypsin was heat inactivated for 5 mins at 95 °C. To create a sample with low phosphorylation, tryptic peptides were subjected to an in vitro dephosphorylation reaction conducted at 30 °C for 30 min with 1 mM MnCl_2_ and 5 µg of bacterially expressed lambda phosphatase in a 60 µL reaction volume. For the sample with high phosphorylation, lambda phosphatase was excluded from the reaction. Reactions were quenched by the addition of 10 µL of 500 mM EDTA. 15 µL of each sample was injected into the LC-MS three times sequentially to create technical replicates and the average peak areas of all replicates were used to calculate *k* with Eq. 2. The resulting *k* values for all AMPK phosphopeptides are displayed in Table 2. The phosphatase reaction completely dephosphorylated every phosphopeptide (data not shown).

2 $${\rm{Flyability}}\,({\rm{k}})=\frac{\left[{{\rm{I}}}_{{\rm{P}}{\bf{A}}}-\,{{\rm{I}}}_{{\rm{P}}{\bf{B}}}\right]}{\left[{{\rm{I}}}_{{\rm{pP}}{\bf{A}}}-\,{{\rm{I}}}_{{\rm{pP}}{\bf{B}}}\right]}$$

### LC-MS/MS analysis of adenine nucleotides

Cell cultures grown in six-well plates were gently washed in ice cold PBS, lysed with 150 mL of ice cold 0.5 M perchloric acid (Univar) and clarified by centrifugation (16,000 *g*, 3 min, 4 °C). Clarified lysate (75 µL) was neutralised with 25 µL of ice cold 2.3 M KHCO_3_ (Sigma–Aldrich), incubated on ice for 5 min and then centrifuged (16,000 *g*, 3 min, 4 °C). Supernatants were collected for analysis by LC-MS/MS.

Adenine nucleotides were measured as previously described^84^. Briefly, a QTRAP 5500 mass spectrometer (AB Sciex) linked to a Prominence HPLC system (Shimadzu) was controlled and managed with the Analyst 1.7.1 software (AB Sciex). The column oven housed a 150 mm (length) × 0.5 mm (inner diameter) Hypercarb 3 µm porous graphitic carbon column (Thermo Fisher Scientific). The LC solvent system comprised of 50 mM triethylammonium bicarbonate buffer (TEAB, Sigma–Aldrich) pH 8.5 in pump A, and acetonitrile with 0.5% trifluoroacetic acid (TFA; Sigma–Aldrich) in pump B. A flow rate of 400 mL min^−1^ was used throughout a gradient program consisting of 0% B (2 min), 0 to 100% B (10 min), 100% B (3 min), 0% B (2 min). Data was analysed with MultiQuant 3.0.2 software (AB Sciex) using area under the LC curve. Calibration curves were determined using the Wagner quadratic equation (ln *y* = *a*_2_ [ln *x*]^2^ + *a*_1_ [ln *x*] + *a*_0_) using the peak area of each nucleotide and were required to have a correlation coefficient (*R*^2^) of >0.99.

### Cell proliferation assays

Cell proliferation assays were conducted as described previously^32^. HEK293 cells were seeded at ~10–15% confluency 48 h before a double transfection with AMPK α2 and β1 or β2 (COOH-terminal FLAG fusion, pcDNA3.1 vector, wild-type and indicated mutants) using Lipofectamine 2000 (Thermo Fisher Scientific) as per the manufacturer’s protocol. Media was replaced 24 h after transfection with either fresh DMEM (supplemented with 10% FBS, 4 mM l-glutamine and penicillin-streptomycin) or arginine- and lysine-free DMEM (supplemented with dialysed 10% FBS and penicillin-streptomycin). Cell proliferation was tracked in real-time using the Incucyte® Live-Cell Analysis System (Sartorius) according to the manufacturer’s instructions.

### RNA-seq analysis

RNA was isolated from HEK293T WT and β2-S184A KI cells using RNeasy mini kit (Qiagen) and treated with DNaseI. RNAseq libraries were prepared from post-ribosome depleted total RNA with the NEBNext Ultra II Directional RNA Library Prep Kit following the manufacturer’s instructions^85^. The libraries were sequenced as 150 bp paired end reads by Novogene (Singapore) on the Illumina platform.

### Gene expression

For transcriptome analysis, reads were trimmed with fastp^86^. Trimmed reads were aligned using Salmon^87^ (version v1.10.2) against gencode v40. Differential gene expression analysis was performed using the Degust analysis tool (http://victorian-bioinformatics-consortium.github.io/degust/). Briefly, genes were only considered with count > 10 and CPM > 1 in at least three samples of a given genotype. Normalised read counts (moderated log counts per million) and differential expression were generated using edgeR^88^.

### Statistical analysis

All statistical analyses were performed using Prism v9.2.0 (GraphPad Software). Results from replicate experiments (n) are expressed as means ± standard error (SEM).

## Supplementary information

Supplementary information

## Data Availability

The RNAseq dataset described in this work is deposited in GEO under accession code GSE272077. All materials and models will be made available upon reasonable request.
