# Supplementary material for: AMPK phosphosite profiling by label-free mass spectrometry reveals a multitude of mTORC1-regulated substrates
Source: NPJ Metab Health Dis. 2025 Mar 4;3:8. doi: 10.1038/s44324-025-00052-7 (PMC11879883; doi:10.1038/s44324-025-00052-7)

# Supplemental File: Uncropped immunoblot images

## Relating to Figure 2A

AMPK α-pT172

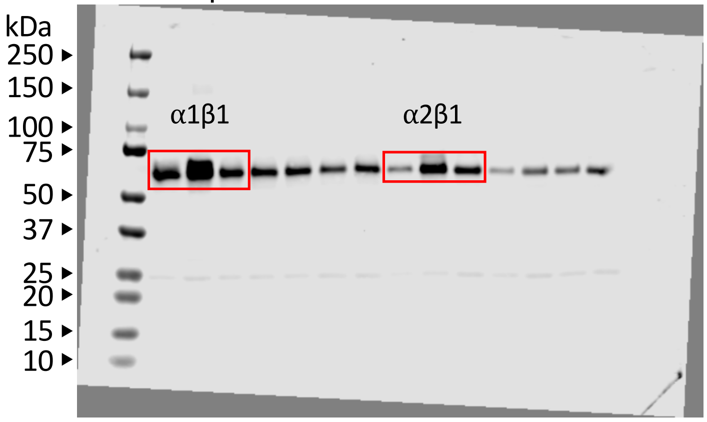


AMPK α-pT172

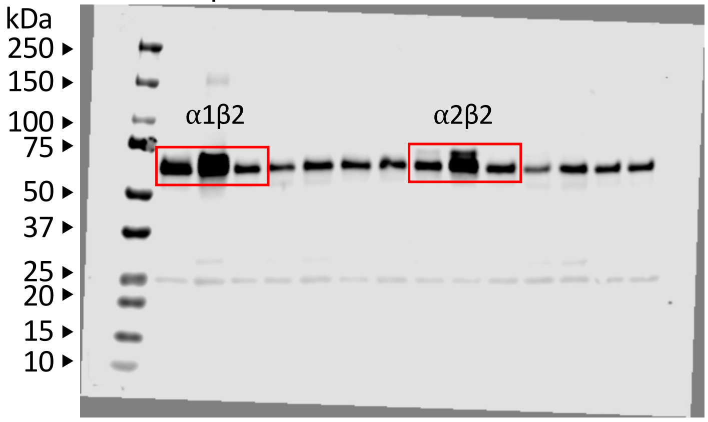


AMPK α-pS487/491

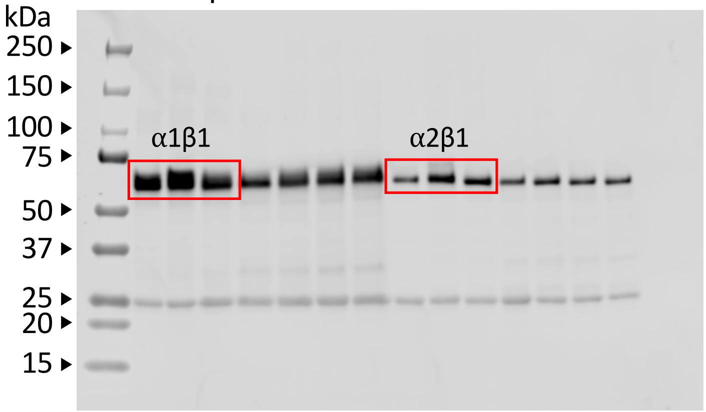


AMPK α-pS487/491

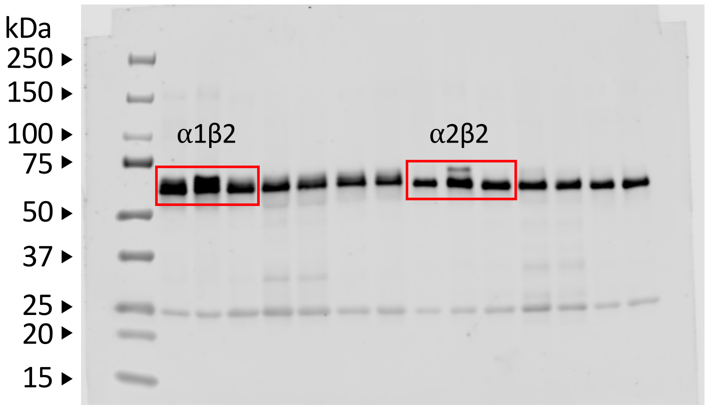


AMPK α

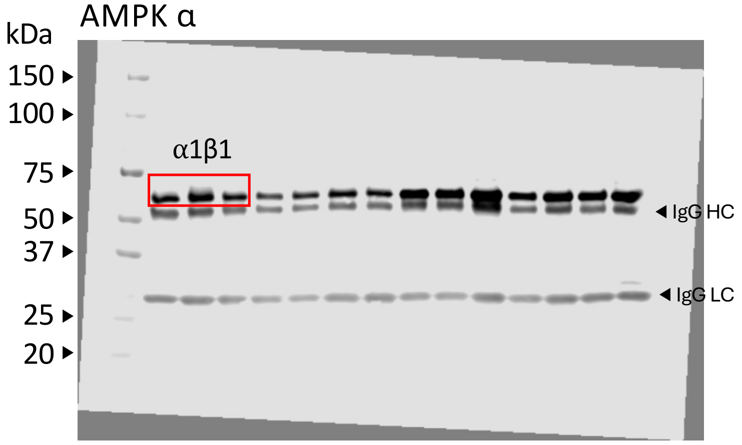


AMPK α

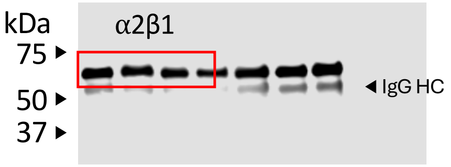


AMPK α

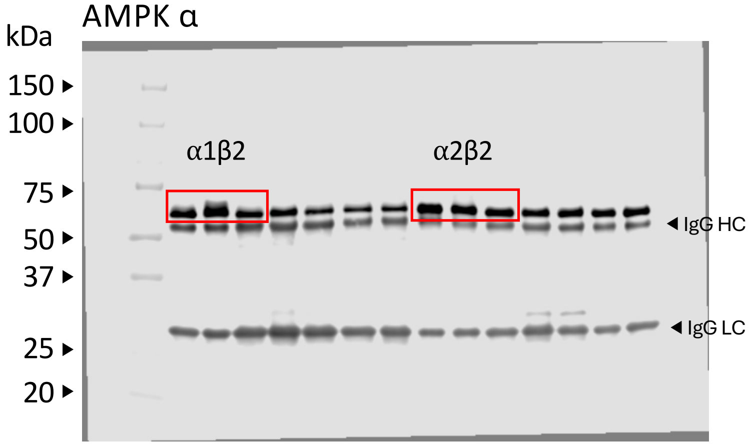

**Relating to Figure 2A**

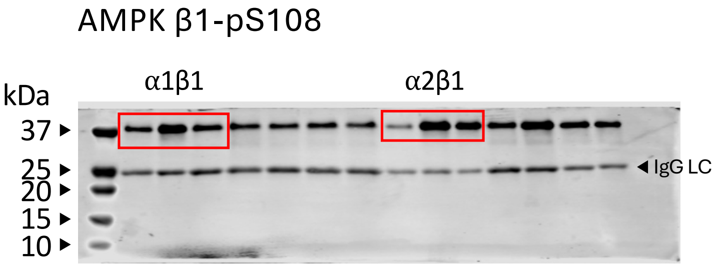


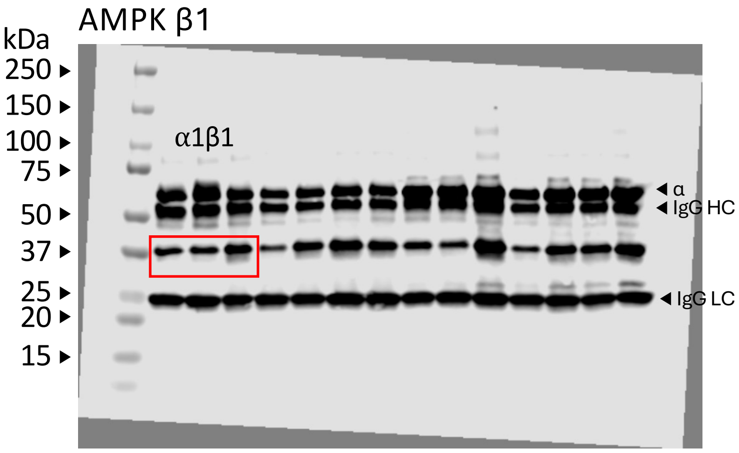


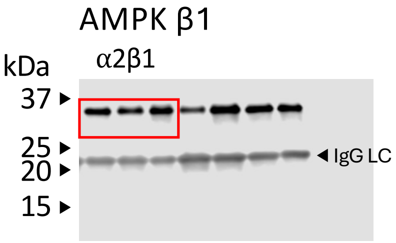


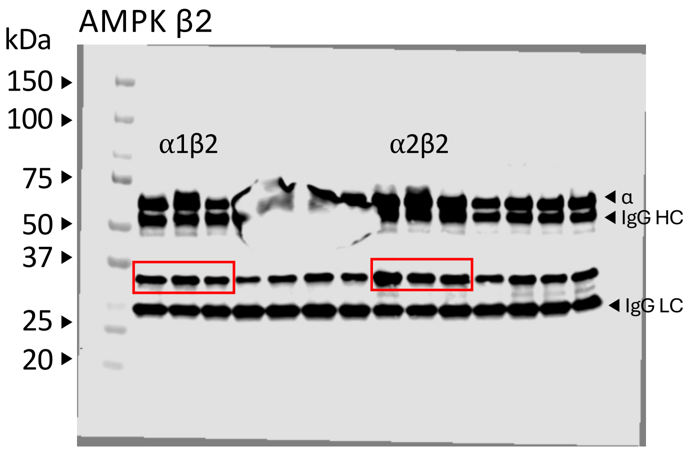


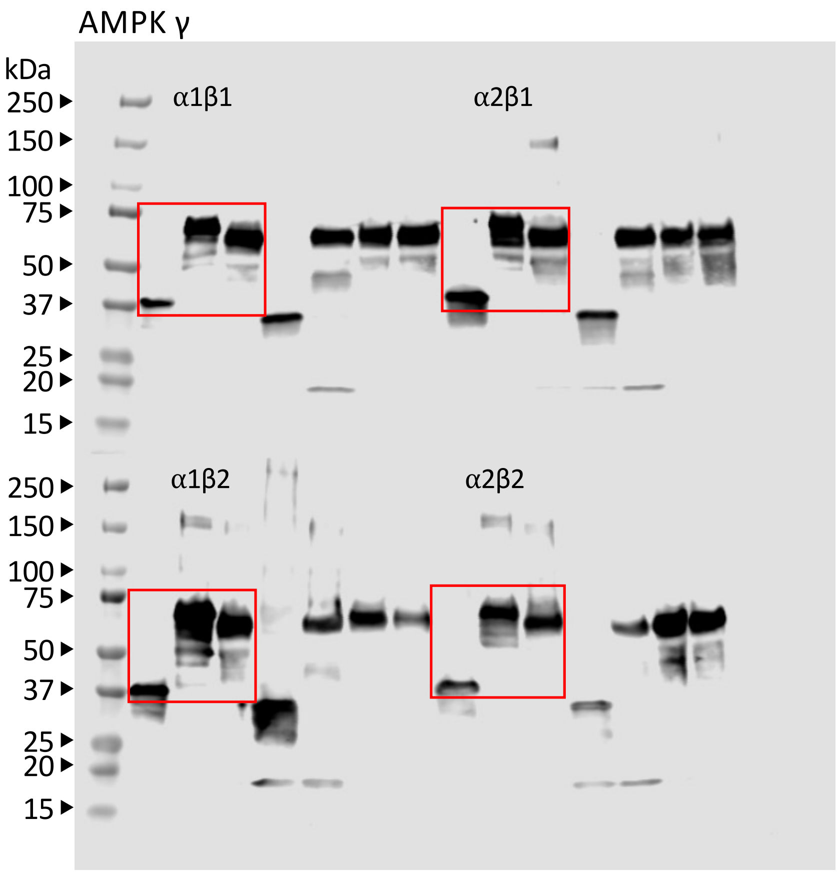

**Relating to Figure 3B**

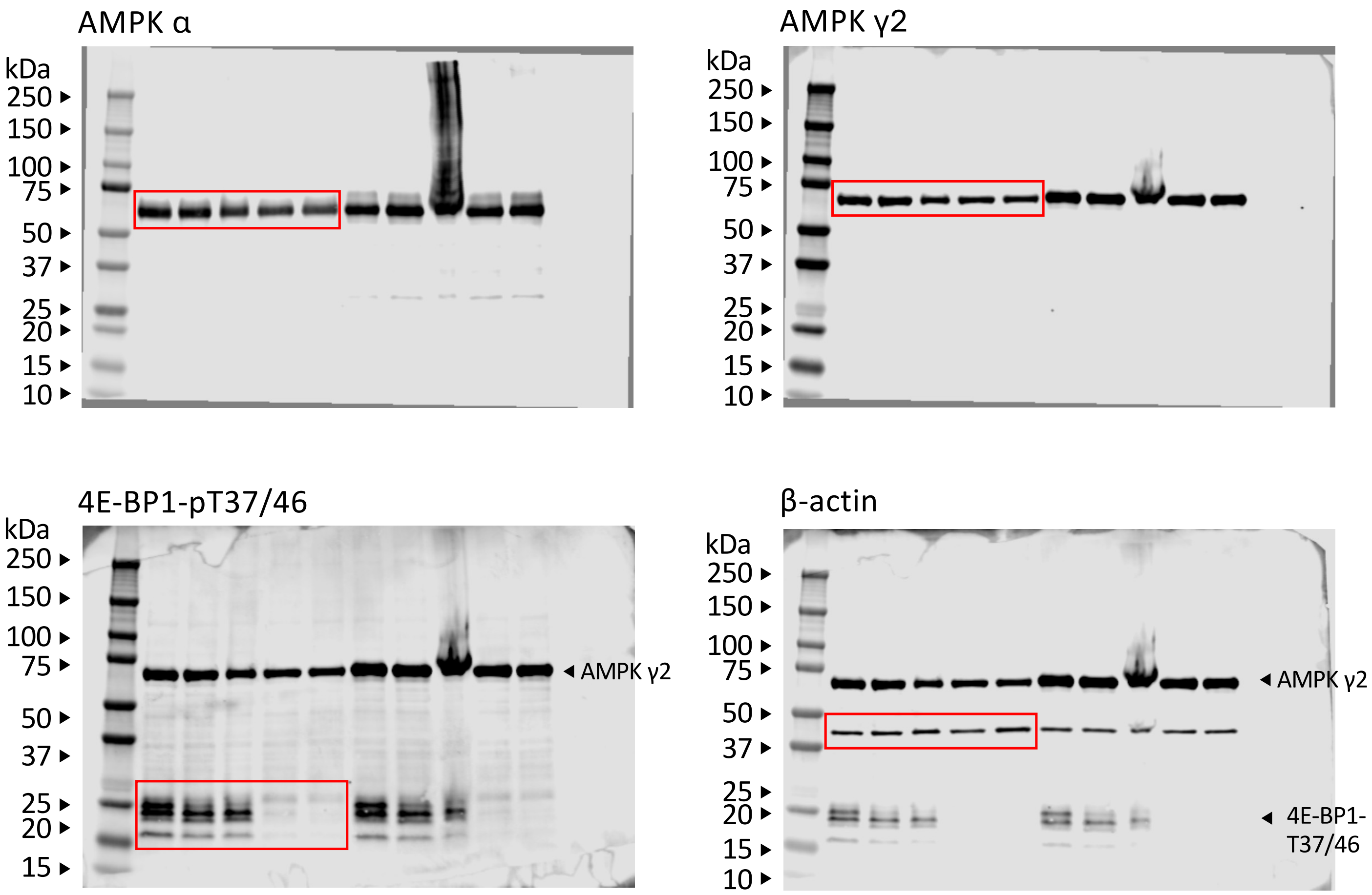

**Relating to Figure 4A**

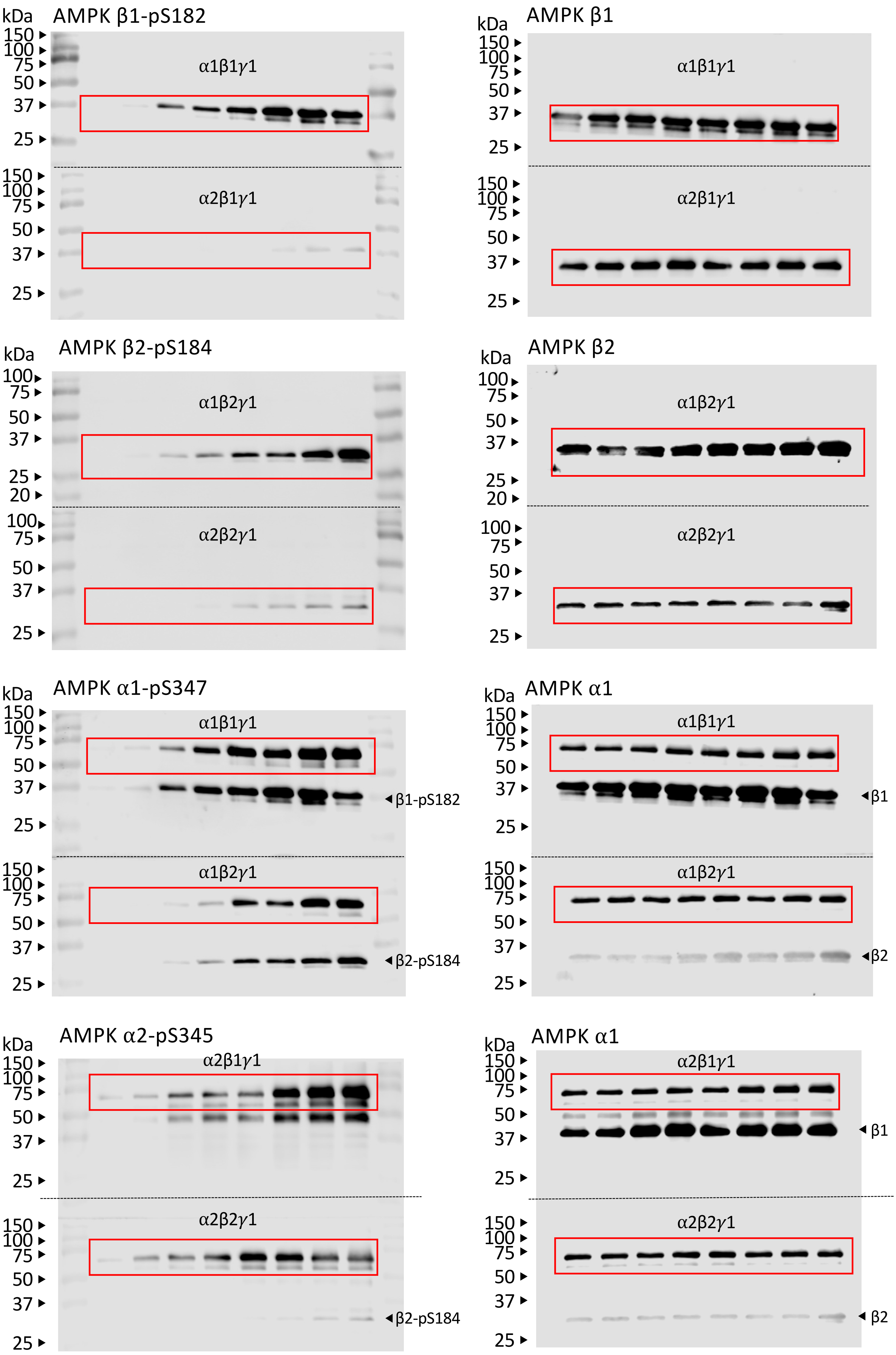

**Relating to Figure 4C & 4D**

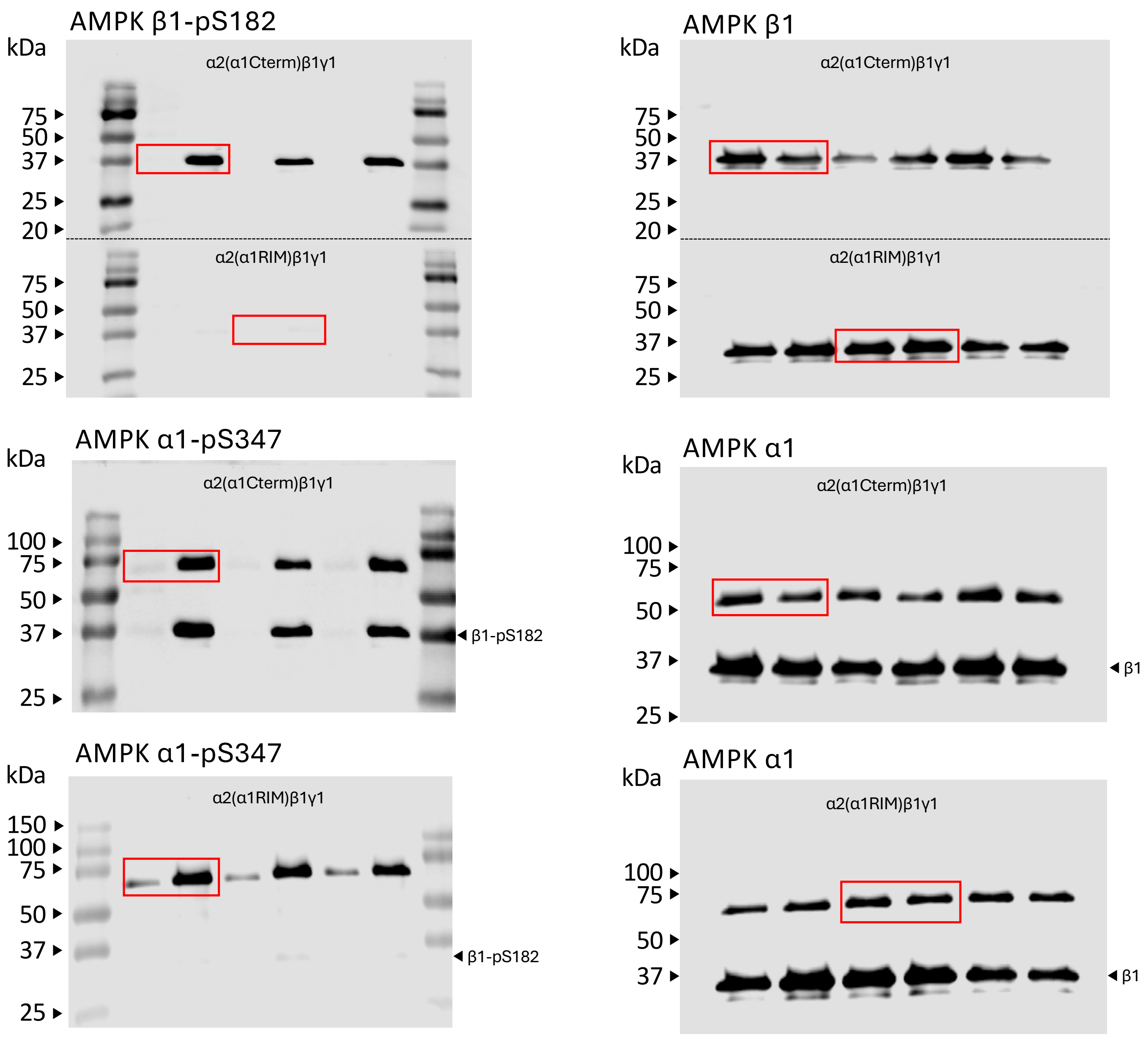

**Relating to Figure 4E**

AMPK β1-pS182

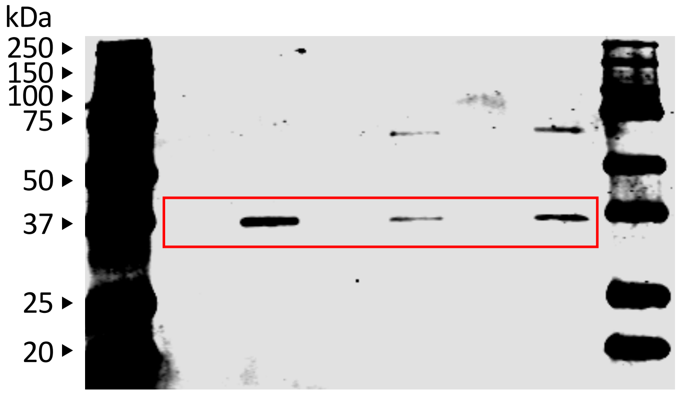


AMPK β1

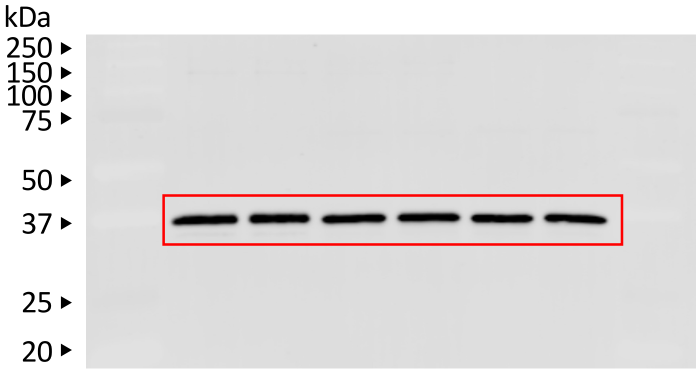


**Relating to Figure 4F**

AMPK α-pT172

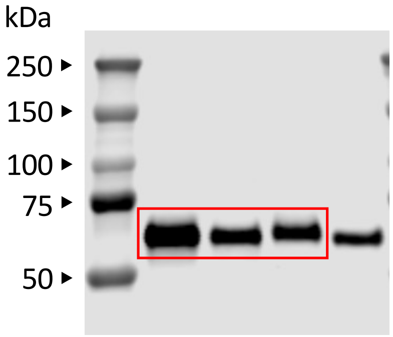


AMPK α-pS487/491

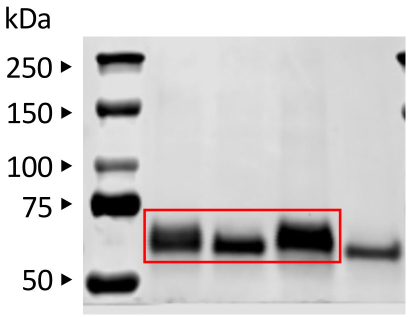


AMPK α

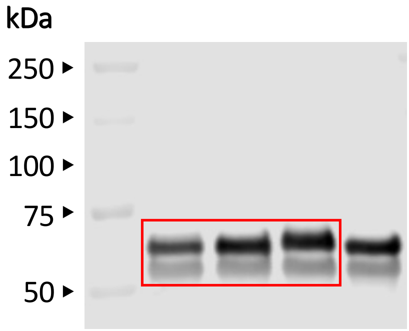


AMPK β2-pS184

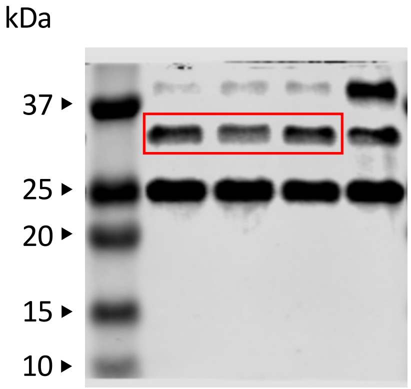


AMPK β2

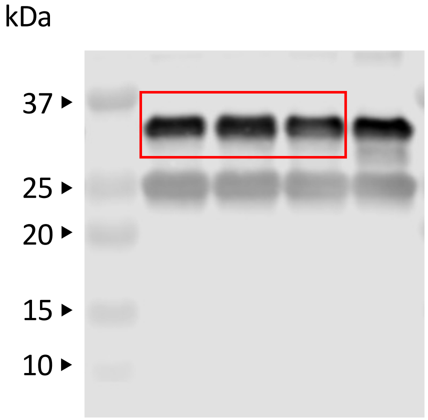


AMPK γ3

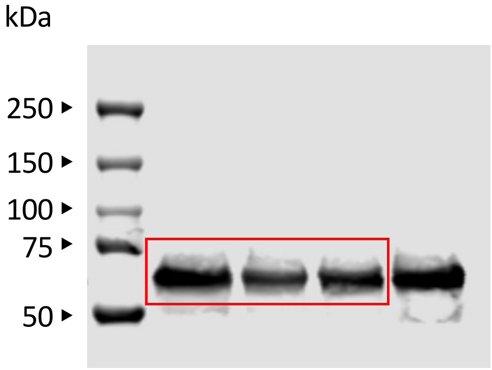

**Relating to Figure 4H**

AMPK β2-pS184

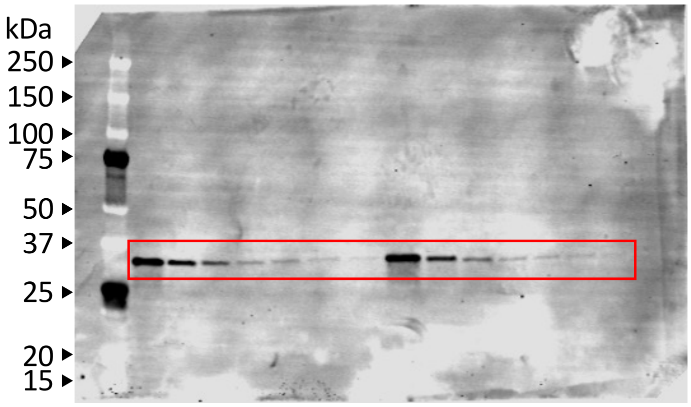


AMPK β2

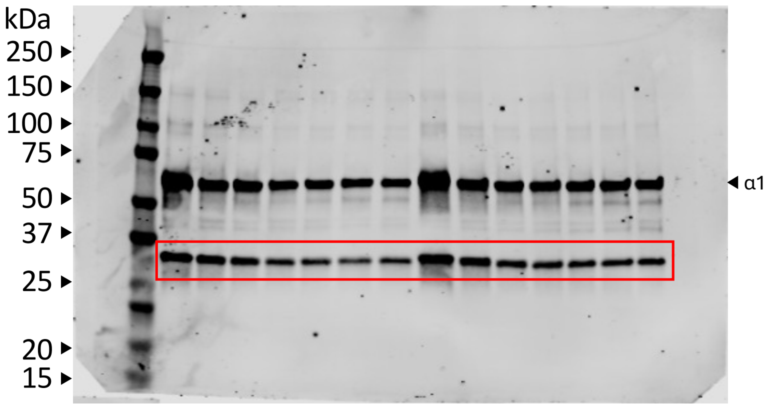


AMPK α1-pT172

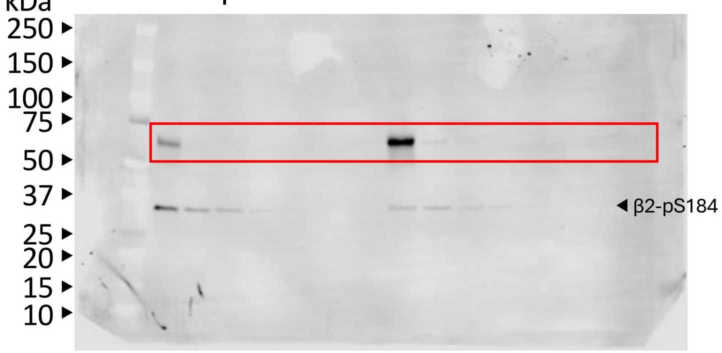


AMPK α1

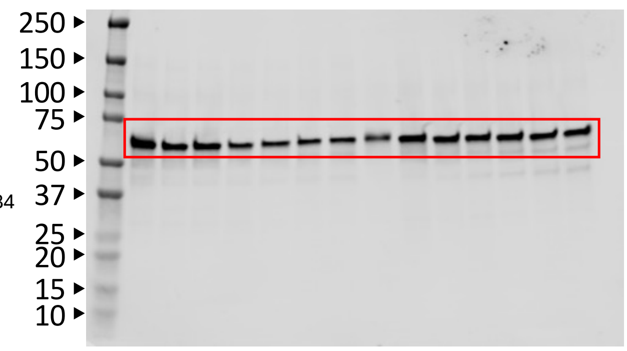


**Relating to Figure 4I**

AMPK β2-pS184

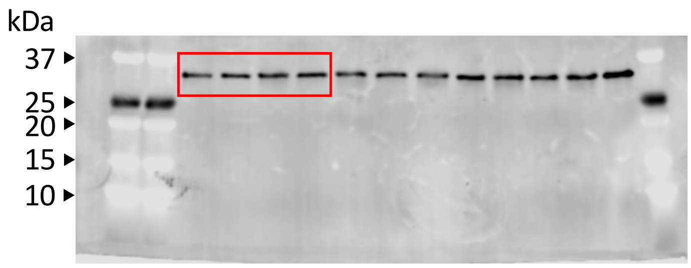


AMPK β2

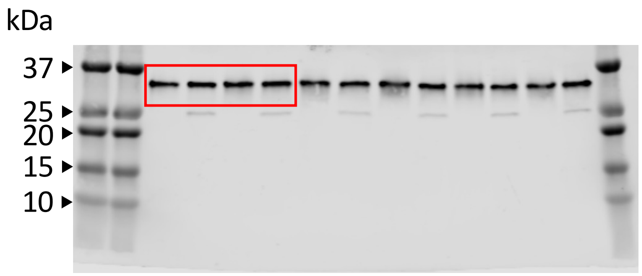


AMPK α1-pT172

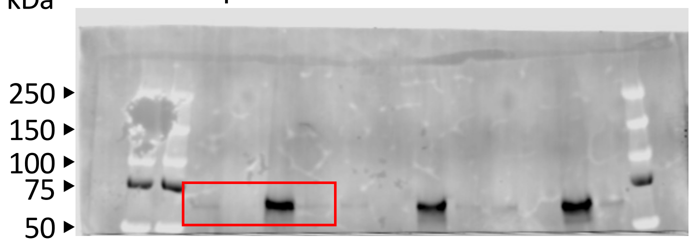


AMPK α1

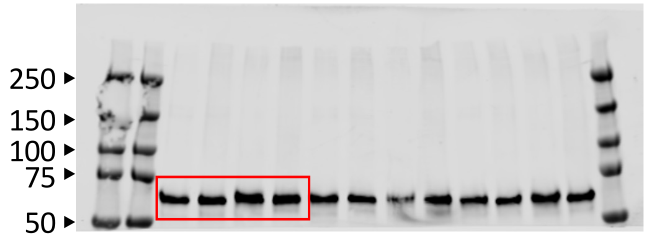

## Relating to Figure 5A

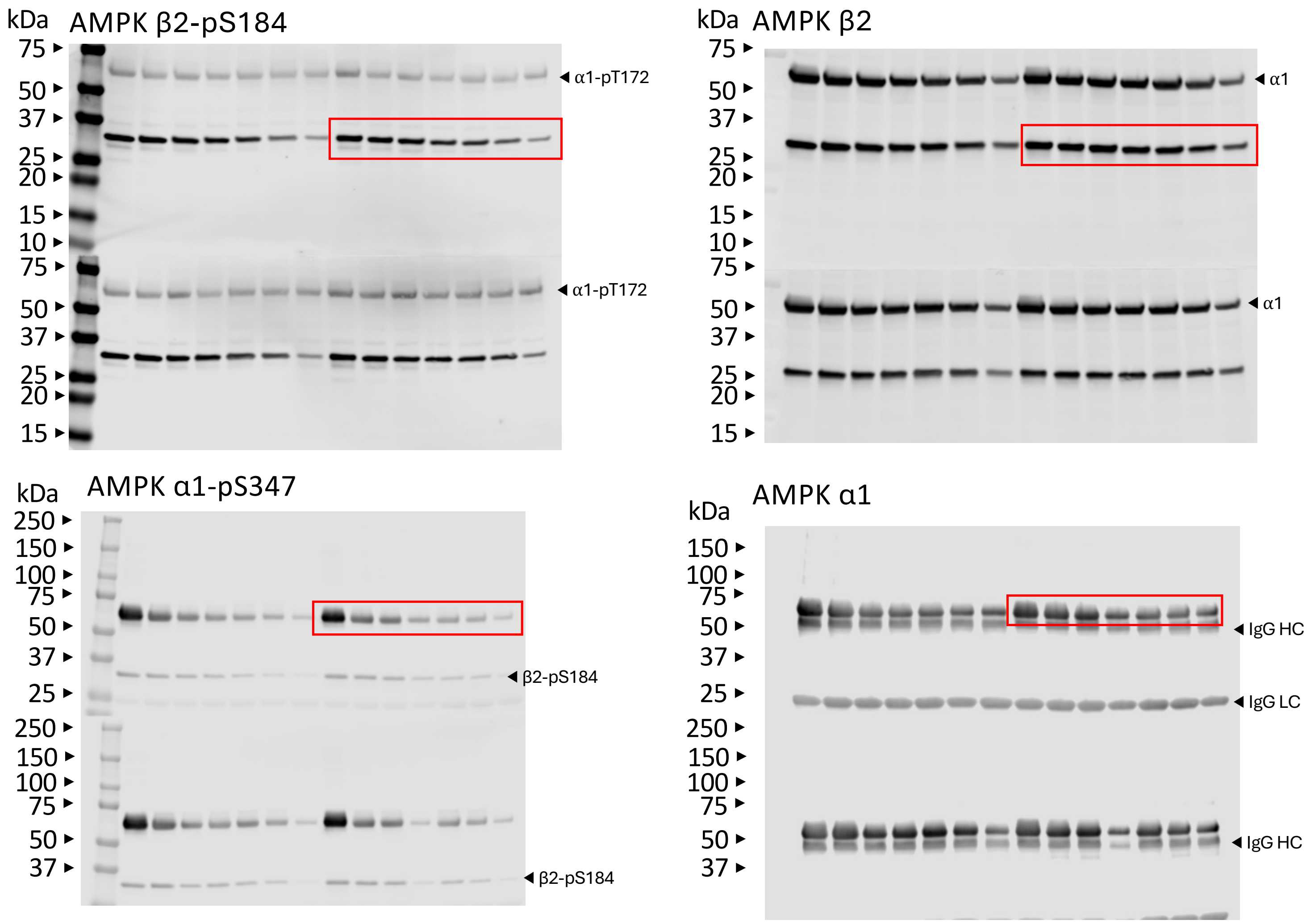


## Relating to Figure 5B

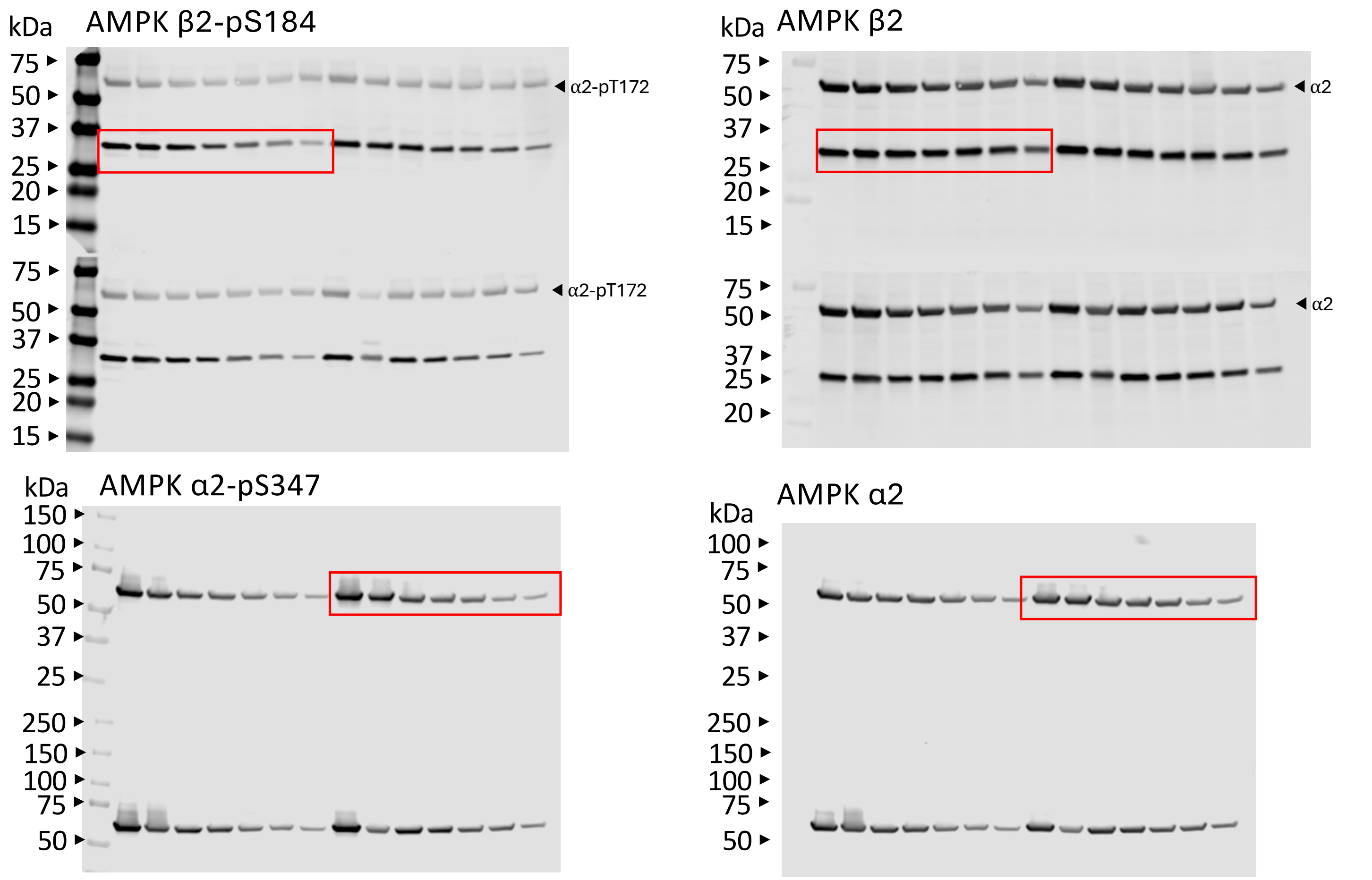

**Relating to Figure 5C**

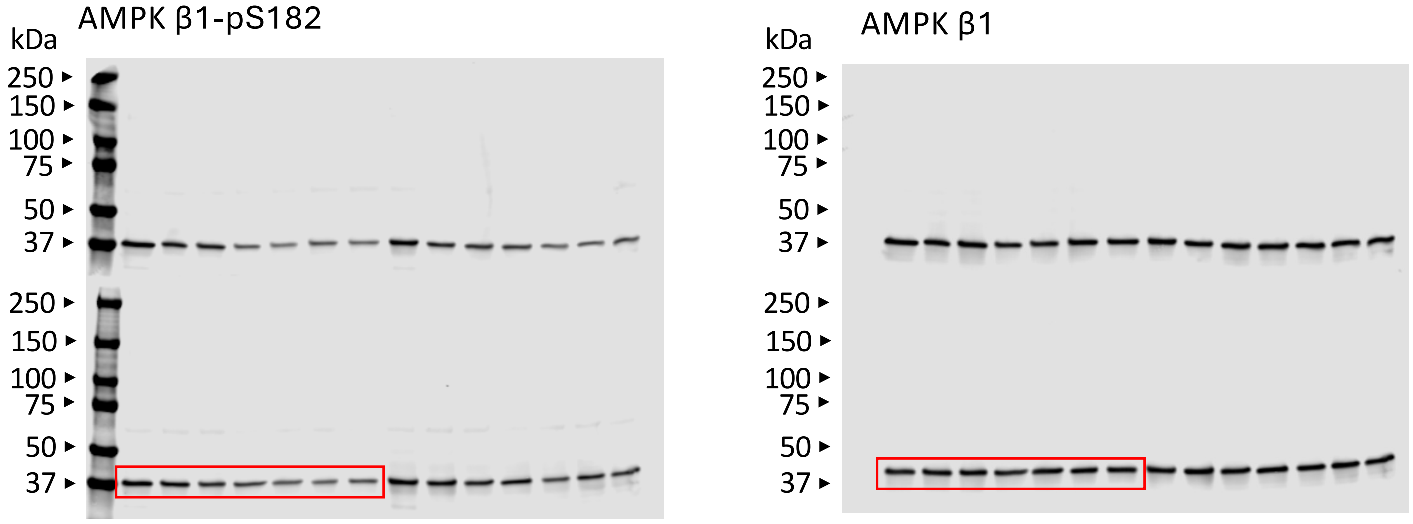


**Relating to Figure 5D**

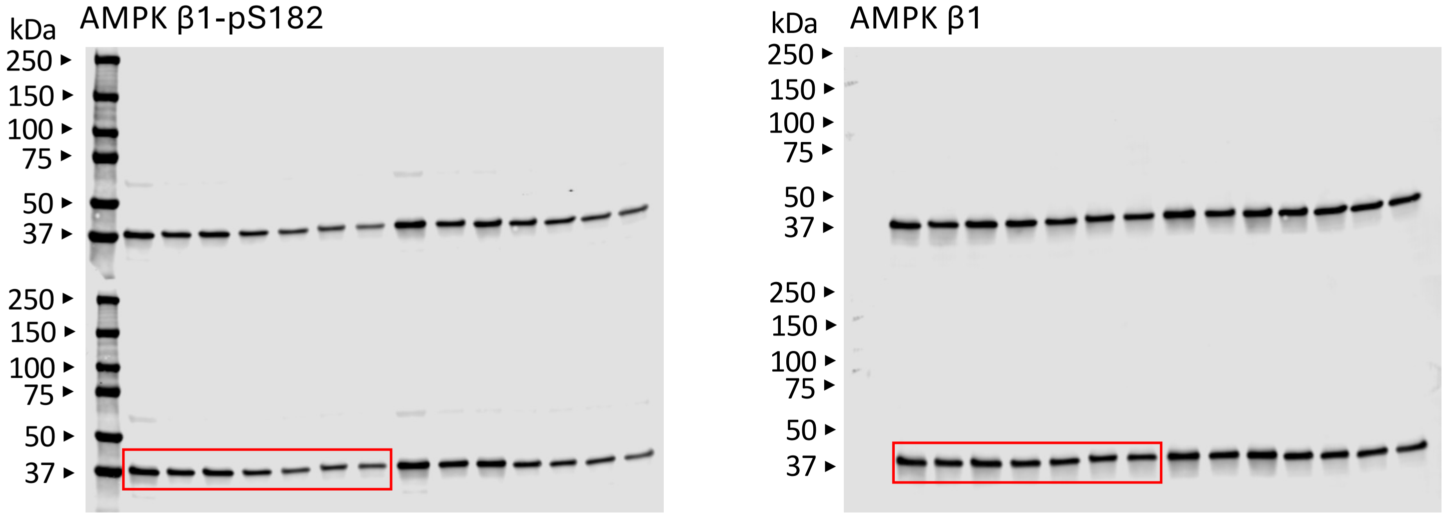

**Relating to Figure 5E**

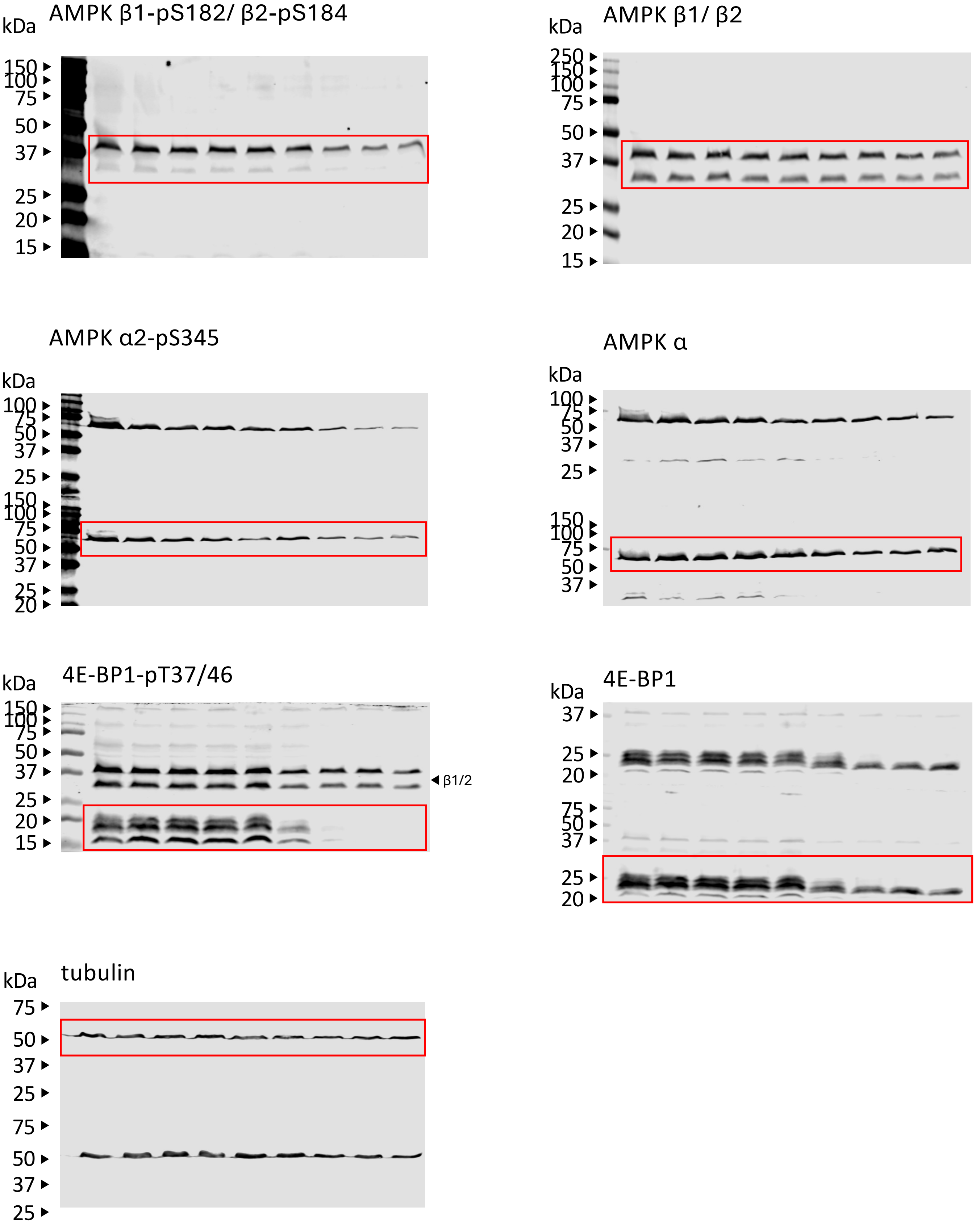

**Relating to Figure 5F**

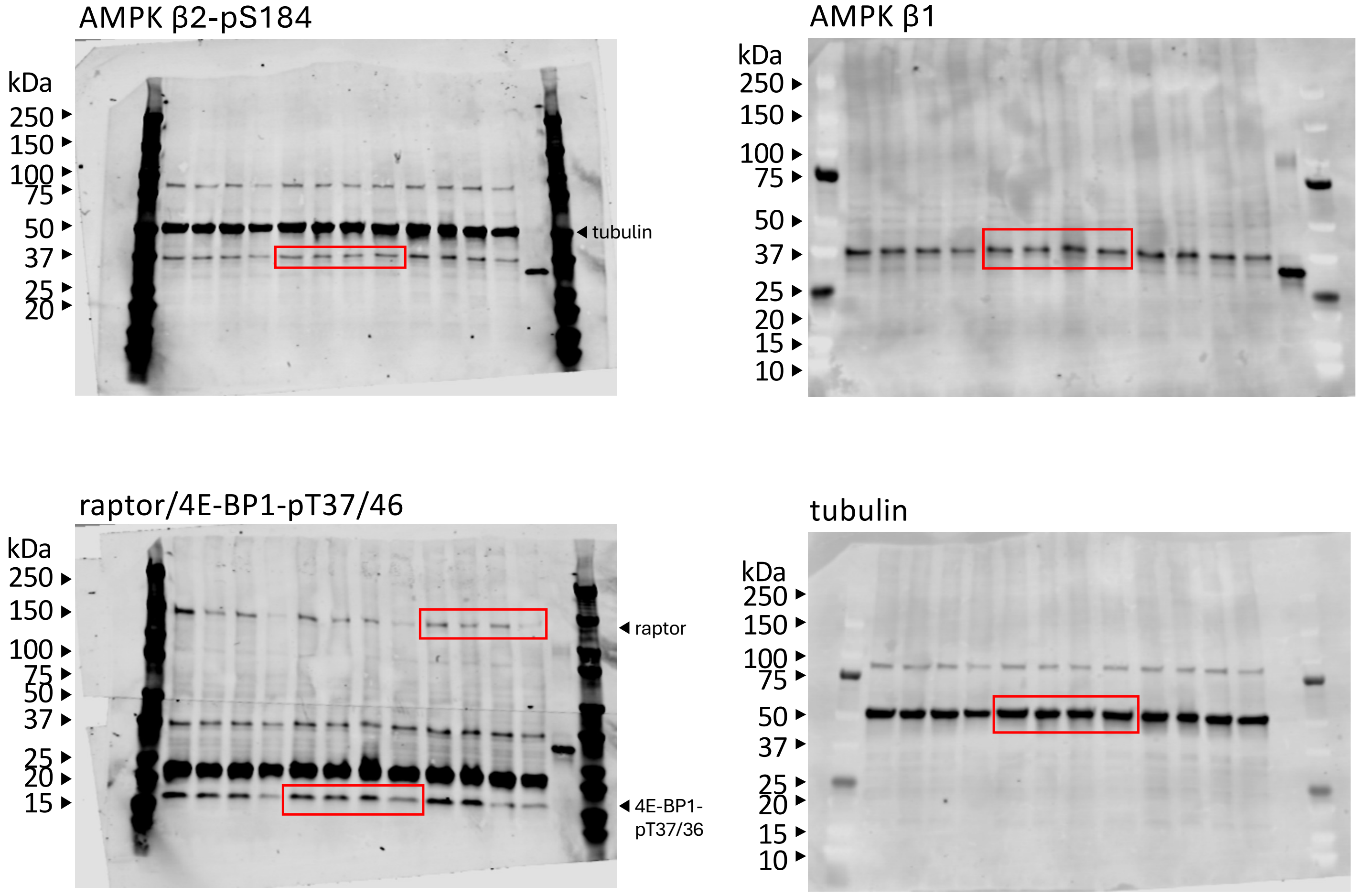


**Relating to Figure 5G**

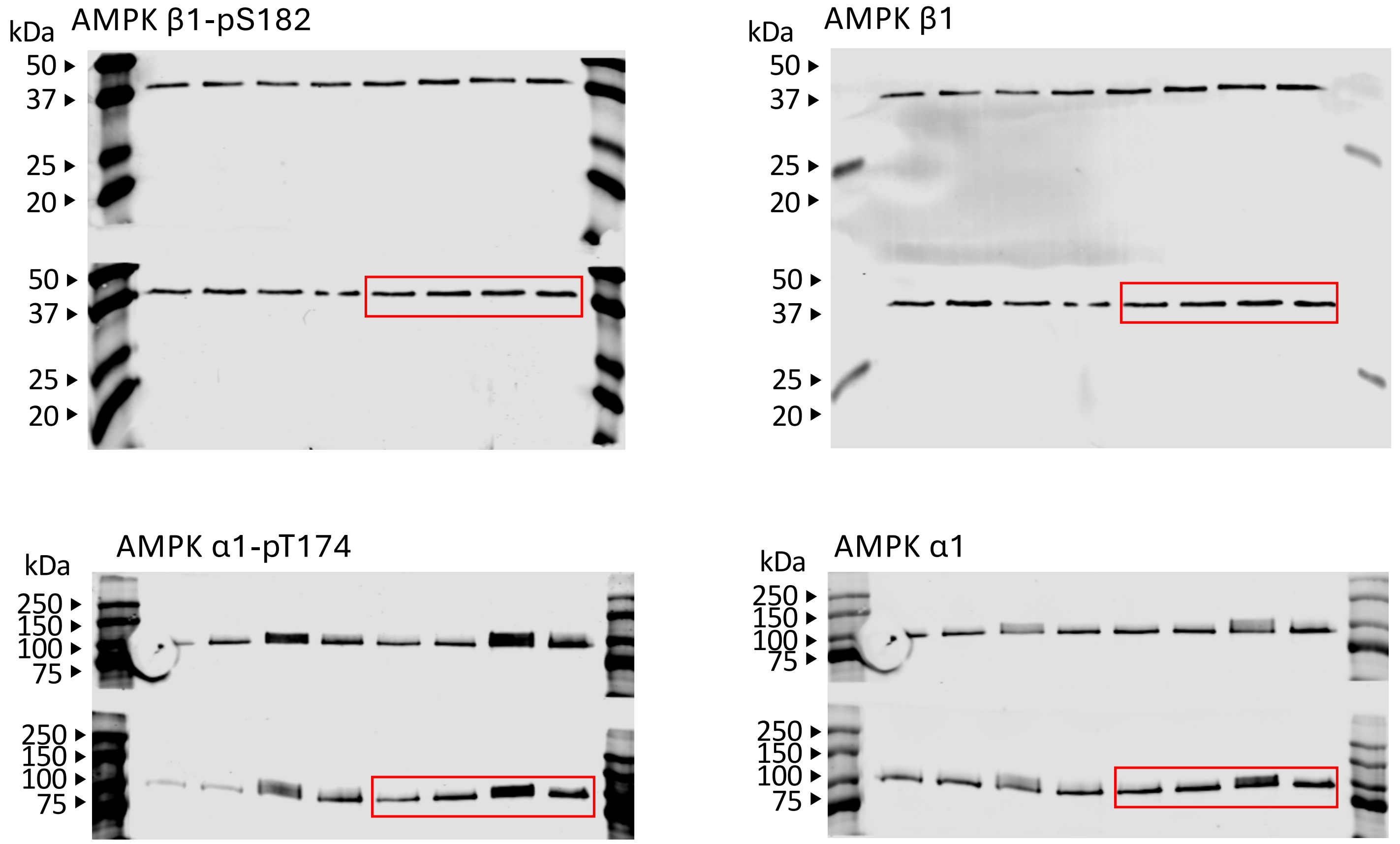

**Relating to Figure 5H**

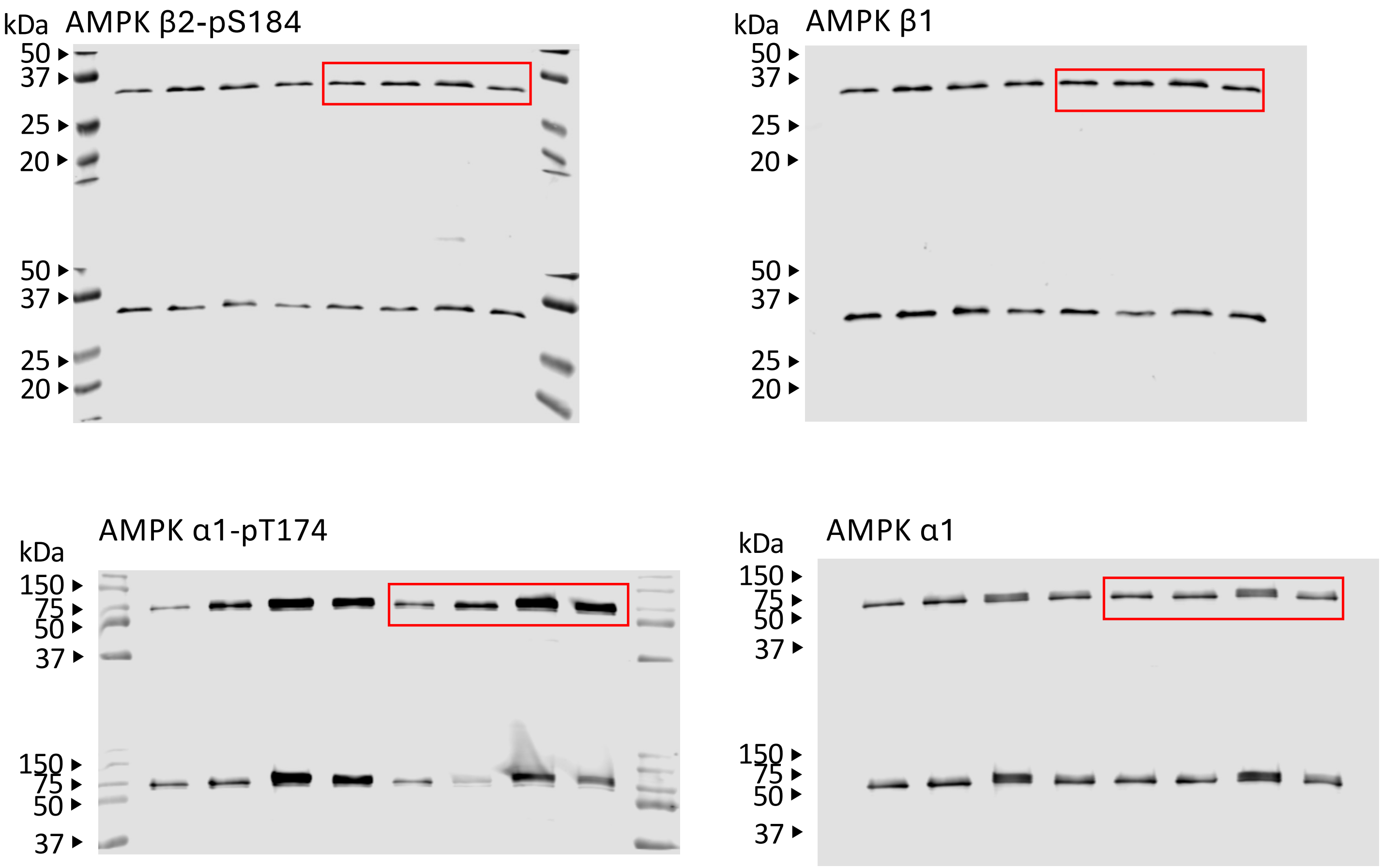

**Relating to Figure 5I**

AMPK β1-pS182

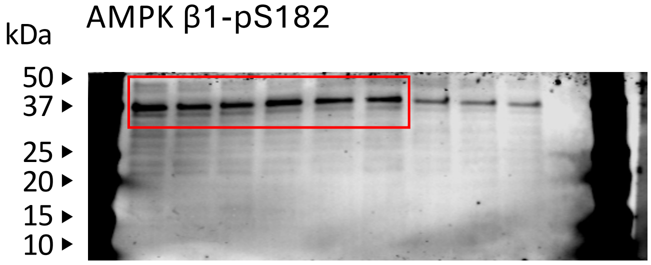


AMPK β1

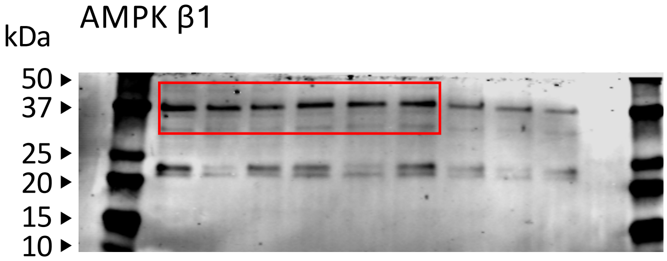


Akt-pS473

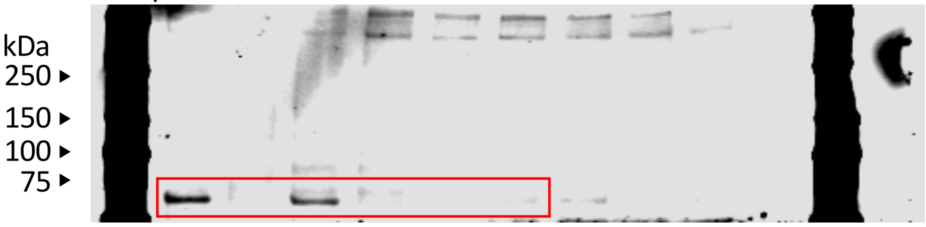


Akt

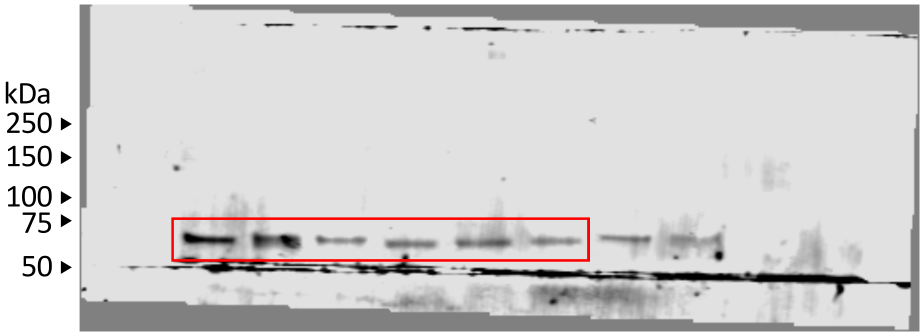

**Relating to Figure 6A**

AMPK β-pS182/184

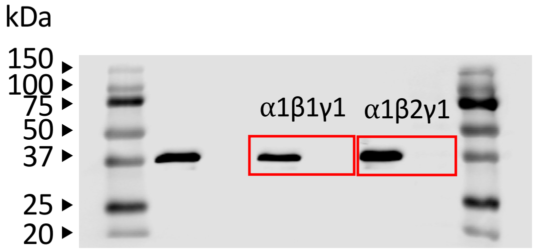


AMPK α-pT172

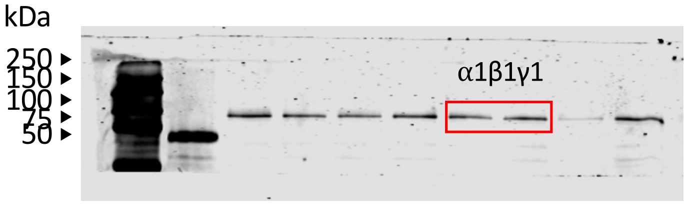


AMPK α-pT172

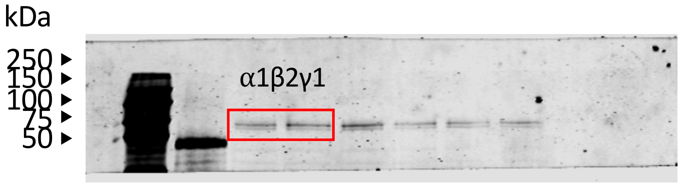


AMPK α1

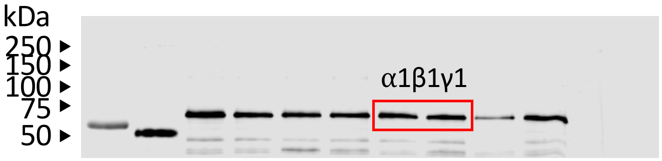


AMPK α1

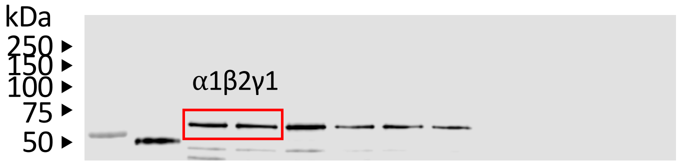


**Relating to Figure 6E**

FLAG-β1

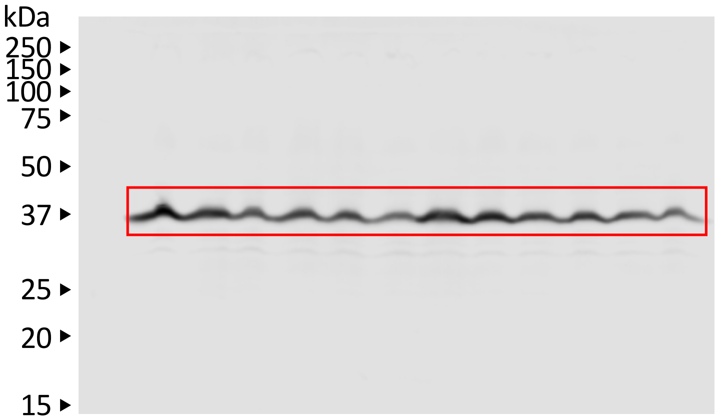


pan-actin/β1-pS182

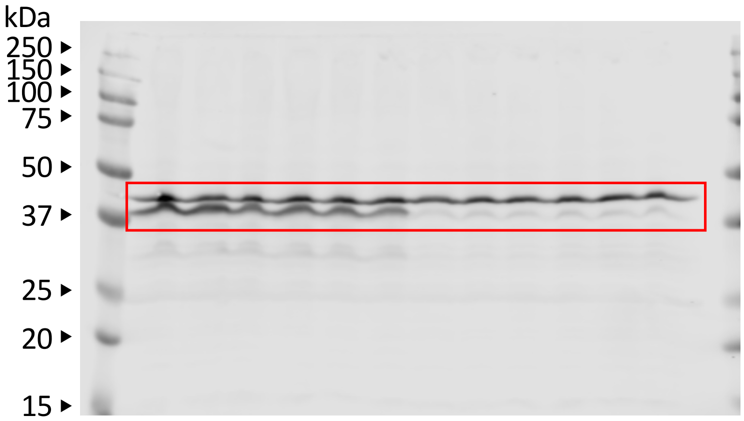

**Relating to Figure 7B**

AMPK β1

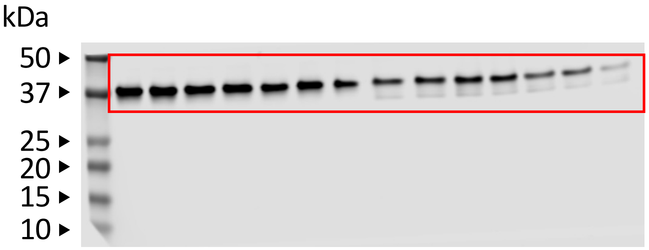


AMPK α2

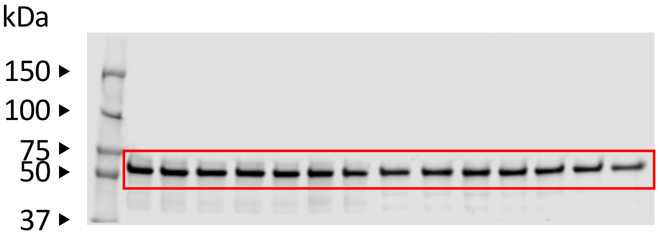


AMPK β1-pS182

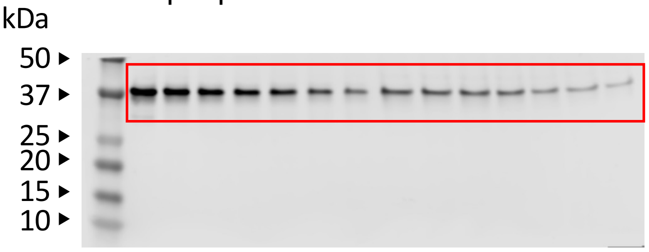


ULK1-pS757

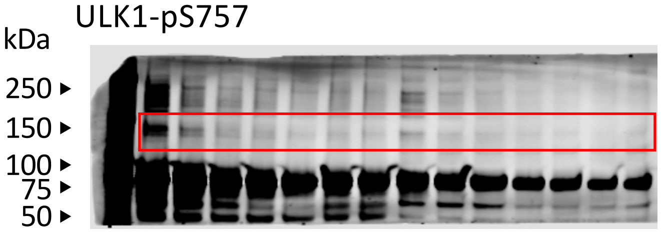


4E-BP1-pT37/46

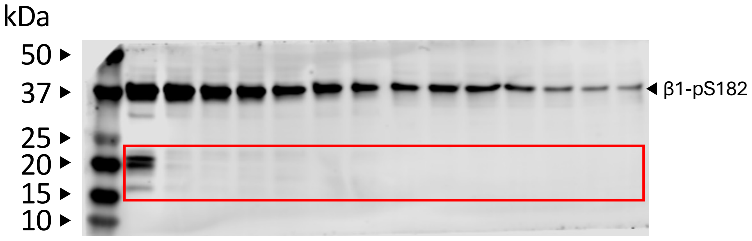


4E-BP1

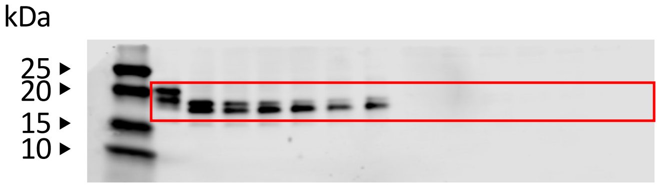

**Relating to Figure 7C**

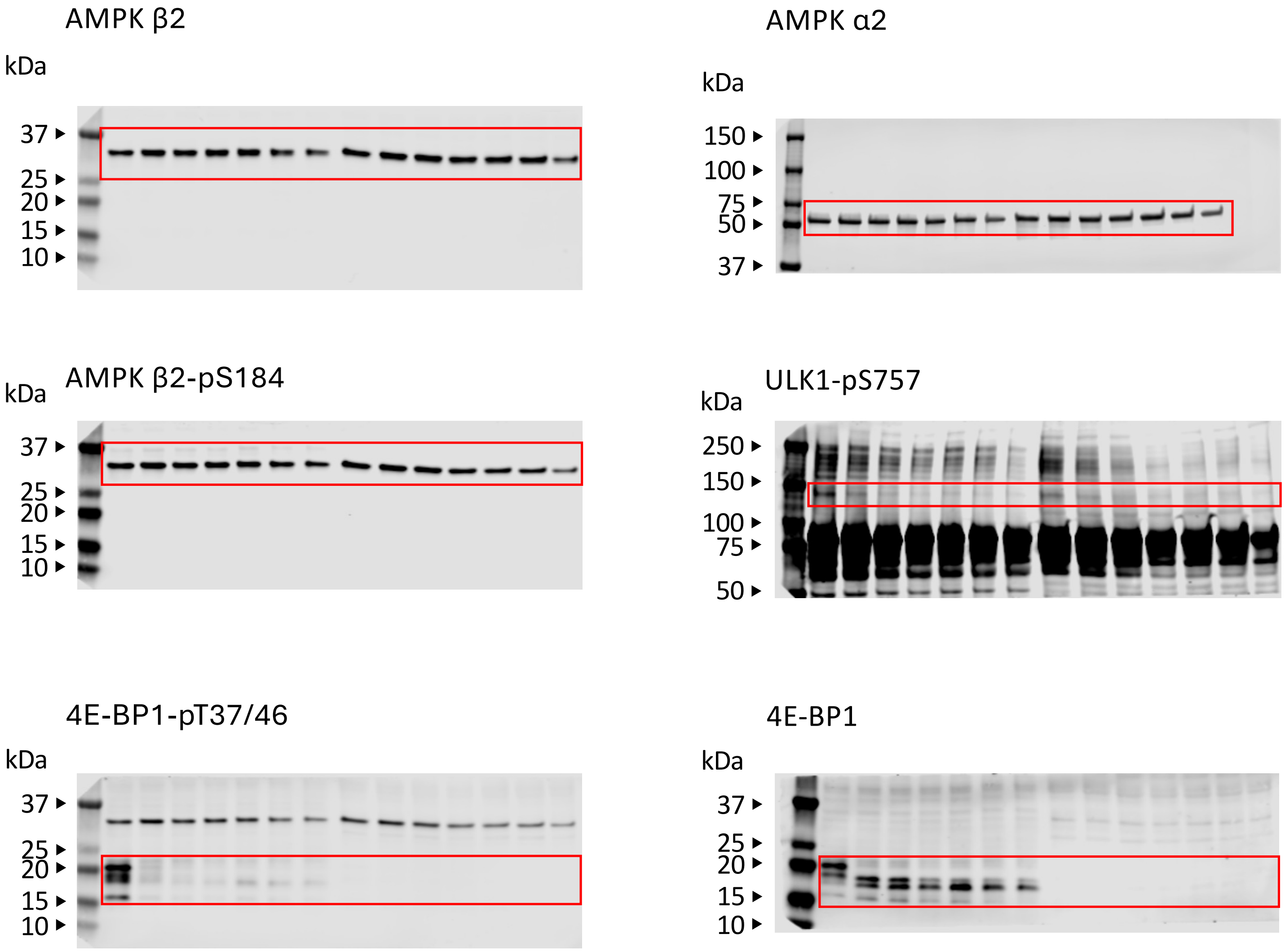

**Relating to Figure 7H**

AMPK β-pS182/184

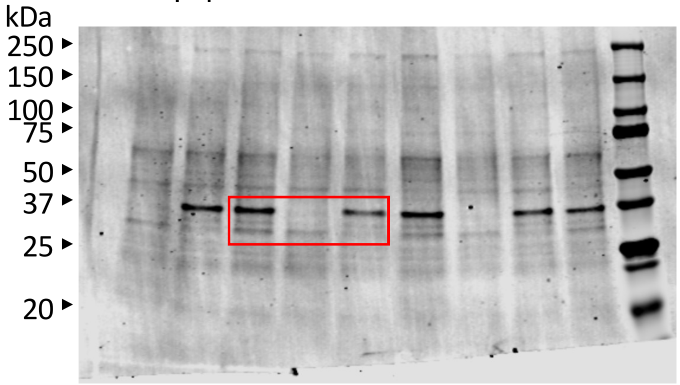


GAPDH

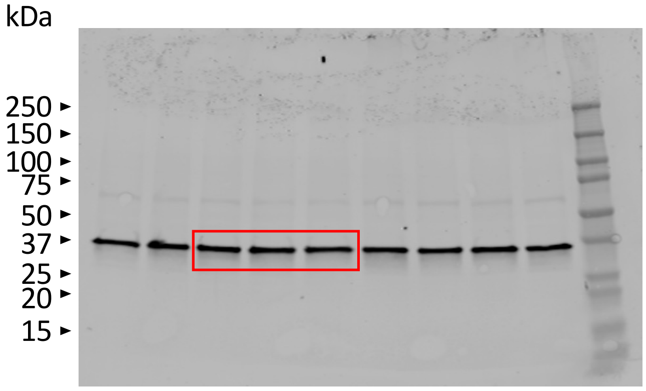


**Relating to Figure 7I**

AMPK α-pT172

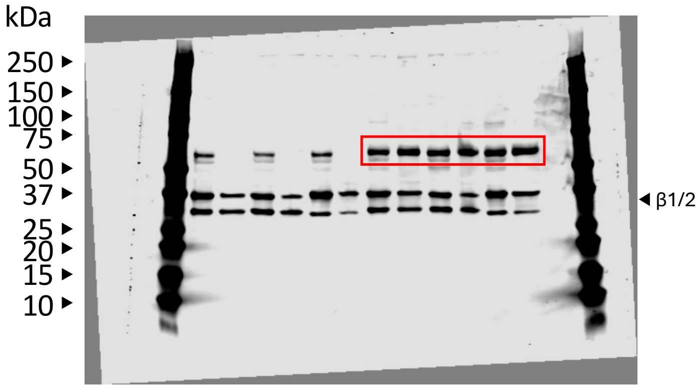


AMPK α

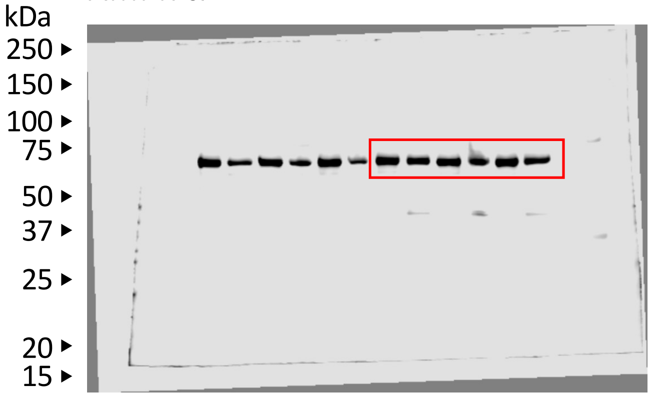


AMPK β-pS182/184

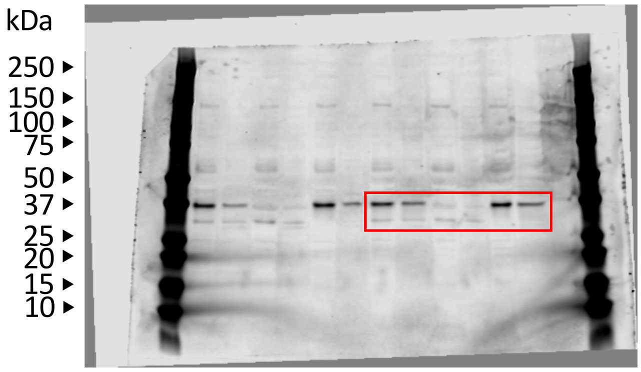


AMPK β1/2

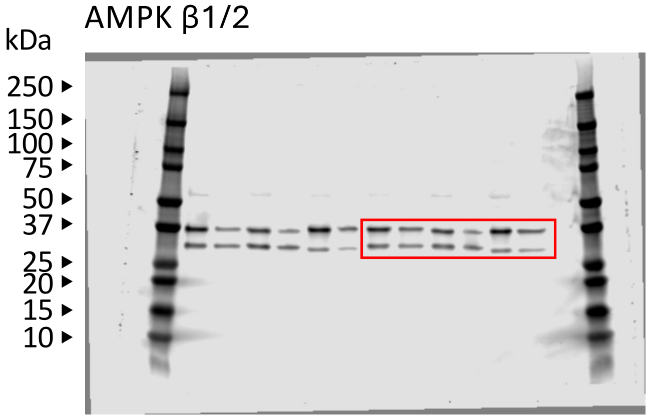


Lamin B1

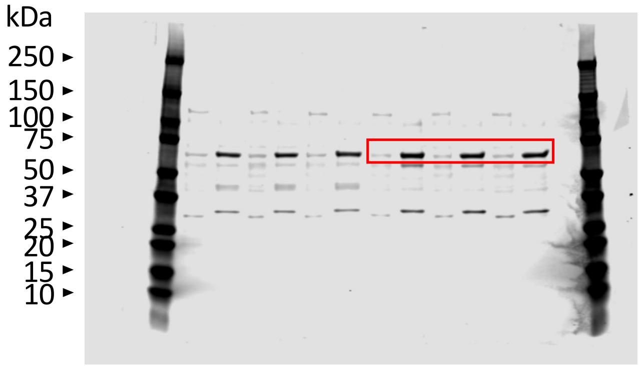


GAPDH

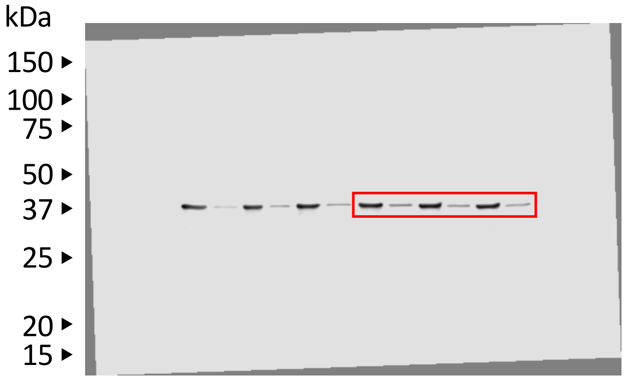

**Relating to Figure 8D**

PCK1

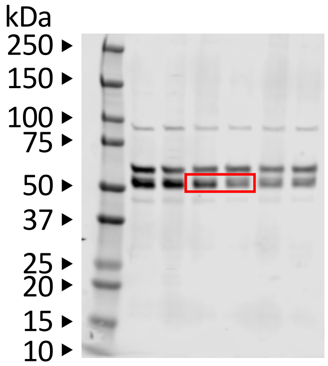


IRS4/GAPDH

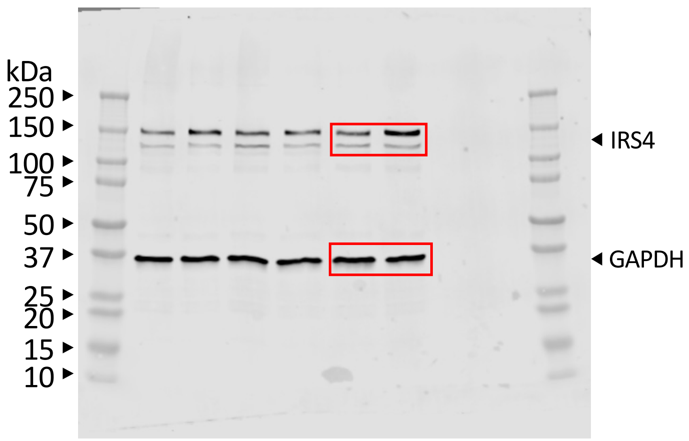


GFPT1

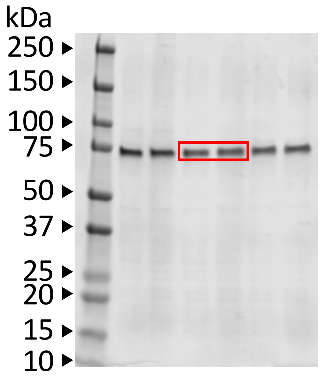


PFKFB3

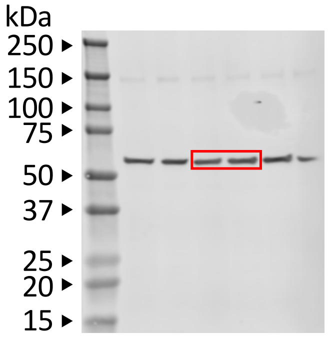


TXNIP

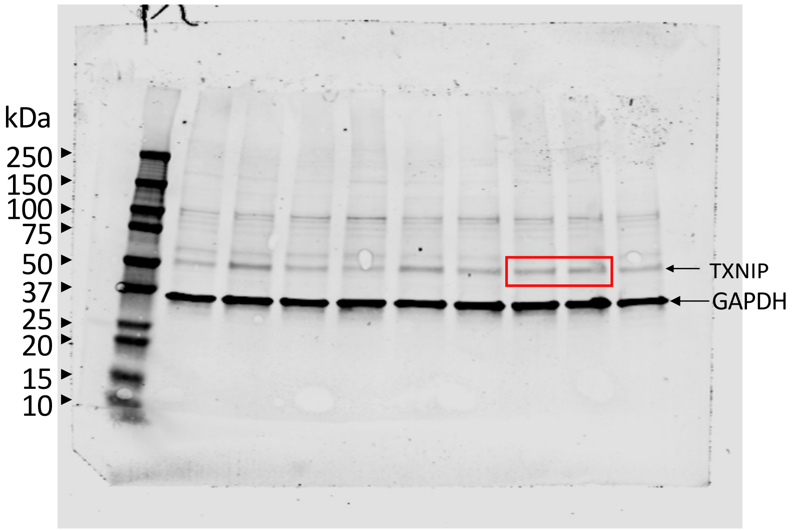


α-tubulin

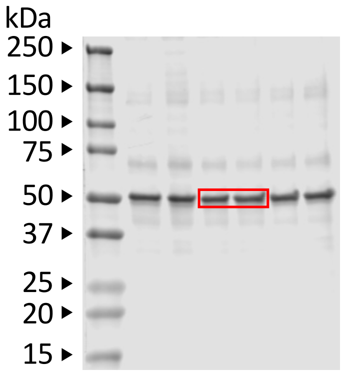

**Relating to Figure 8F**

Akt-pS473

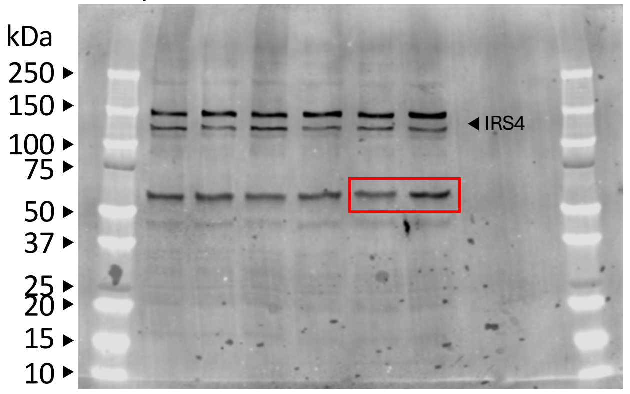


Akt

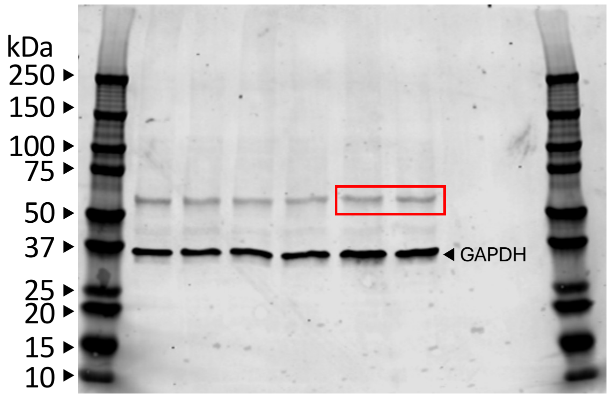


S6K-pT389

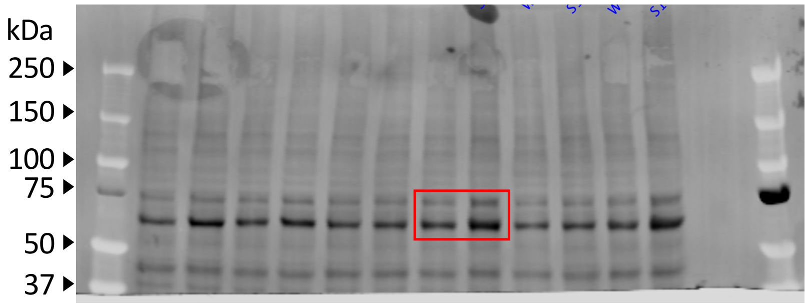


α-tubulin

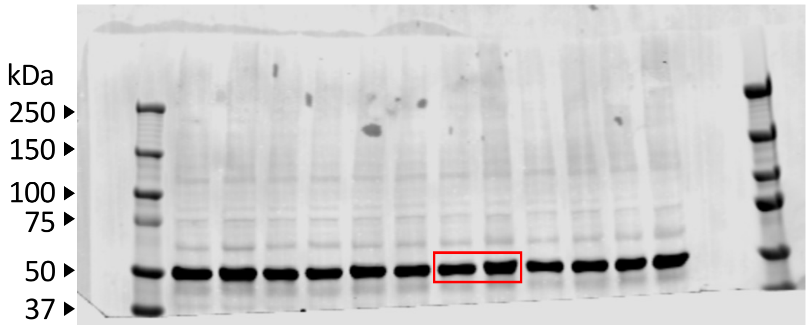

Supplement: Supplementary file 1 — Supplementary information [file 44324_2025_52_MOESM1_ESM.pdf]
